# Tumor-derived miR-6794-5p enhances cancer growth by promoting M2 macrophage polarization

**DOI:** 10.1186/s12964-024-01570-5

**Published:** 2024-03-23

**Authors:** Jae Yeon Choi, Hyun Jeong Seok, Dong Hyeon Lee, Eunju Lee, Tae-Jin Kim, Sangwoo Bae, Incheol Shin, In Hwa Bae

**Affiliations:** 1 Division of Radiation Biomedical Research, Korea Institute of Radiological & Medical Sciences, 75 Nowon-ro, Nowon-gu, Seoul, 01812 Republic of Korea; 2 Department of Life Science, Hanyang University, Seoul, Republic of Korea

**Keywords:** Exosomal miR-6794-5p, Tumor malignancy, Macrophage M2 polarization, SOCS1, IL-10, Tumor microenvironment

## Abstract

**Background:**

Solid tumors promote tumor malignancy through interaction with the tumor microenvironment, resulting in difficulties in tumor treatment. Therefore, it is necessary to understand the communication between cells in the tumor and the surrounding microenvironment. Our previous study revealed the cancer malignancy mechanism of Bcl-w overexpressed in solid tumors, but no study was conducted on its relationship with immune cells in the tumor microenvironment. In this study, we sought to discover key factors in exosomes secreted from tumors overexpressing Bcl-w and analyze the interaction with the surrounding tumor microenvironment to identify the causes of tumor malignancy.

**Methods:**

To analyze factors affecting the tumor microenvironment, a miRNA array was performed using exosomes derived from cancer cells overexpressing Bcl-w. The discovered miRNA, miR-6794-5p, was overexpressed and the tumorigenicity mechanism was confirmed using qRT-PCR, Western blot, invasion, wound healing, and sphere formation ability analysis. In addition, luciferase activity and Ago2-RNA immunoprecipitation assays were used to study the mechanism between miR-6794-5p and its target gene SOCS1. To confirm the interaction between macrophages and tumor-derived miR-6794-5p, co-culture was performed using conditioned media. Additionally, immunohistochemical (IHC) staining and flow cytometry were performed to analyze macrophages in the tumor tissues of experimental animals.

**Results:**

MiR-6794-5p, which is highly expressed in exosomes secreted from Bcl-w-overexpressing cells, was selected, and it was shown that the overexpression of miR-6794-5p increased migratory ability, invasiveness, and stemness maintenance by suppressing the expression of the tumor suppressor SOCS1. Additionally, tumor-derived miR-6794-5p was delivered to THP-1-derived macrophages and induced M2 polarization by activating the JAK1/STAT3 pathway. Moreover, IL-10 secreted from M2 macrophages increased tumorigenicity by creating an immunosuppressive environment. The in vitro results were reconfirmed by confirming an increase in M2 macrophages and a decrease in M1 macrophages and CD8+ T cells when overexpressing miR-6794-5p in an animal model.

**Conclusions:**

In this study, we identified changes in the tumor microenvironment caused by miR-6794-5p. Our study indicates that tumor-derived miR-6794-5p promotes tumor aggressiveness by inducing an immunosuppressive environment through interaction with macrophage.

**Supplementary Information:**

The online version contains supplementary material available at 10.1186/s12964-024-01570-5.

## Background

The interaction between the tumor and the microenvironment suppresses the immune response by secreting various growth factors and cytokines and is continuously involved in tumor initiation, growth, invasion, metastasis and reprogramming of therapeutic response [1–4]. The tumor microenvironment is composed of heterogeneous cells, including peripheral blood vessels, immune cells, fibroblasts, signaling molecules, and the extracellular matrix [5, 6]. Macrophages can have a dual impact on cancer by reversibly changing their phenotype in response to external stimuli, thereby suppressing the cytotoxic activity of immune cells or promoting antitumor responses [7]. Accumulation of tumor-associated macrophages (TAMs) in solid tumors is known to be associated with poor patient prognosis and has therefore been recognized as a potential diagnostic and prognostic biomarker for cancer [7, 8]. Therefore, analysis of communication methods and mechanisms between cancer cells and the tumor microenvironment has become an important field in understanding cancer. Our previous studies have demonstrated that Bcl-w, a survival-promoting member of the Bcl-2 protein family, is highly expressed in several solid tumors (e.g., GBM, breast cancer, and non-small cell lung cancer) and is involved in EMT, invasion, migration, and metastasis [9–11]. However, despite the close relationship between solid tumors and the tumor microenvironment, the role of Bcl-w within the tumor microenvironment is not well understood.

MicroRNA (miRNA)  are non-coding RNAs of 20–24 nucleotides. Upon gene expression, they bind to the 3’UTR region of a target gene with a complementary nucleotide sequence to degrade mRNA or inhibit translation into proteins [12]. Therefore, miRNAs expressed in tumors act as oncogenes or tumor suppressors by regulating the expression of target genes and are involved in cancer cell proliferation, apoptosis, invasion, and angiogenesis [13]. For example, miR-302d and miR-16 prevent GBM tumorigenesis by directly inhibiting NF-κB and FGF2 [14]. MiR-124 and miR-137 inhibit proliferation by repressing CDK6 expression, leading to G0/G1 cycle arrest [15]. It has been reported that upregulated miR-21 in lung cancer patients involved in cancer growth by suppressing the expression of PTEN [16, 17].

Exosomes are 30–120 nm cell membrane-derived endoplasmic reticulum, which are secreted from various cells including cancer cells, and contain lipids, proteins, mRNAs, and miRNAs [18, 19]. Additionally, because exosomes are fused to the cell membrane of peripheral or distant cells and serve to deliver various biomolecules, they are recognized as important transmitters in intercellular signaling [18–20]. Exosomes are involved in tumor progression, angiogenesis, and metastasis by delivering cytokines, chemokines, growth factors, miRNAs, and various molecules to the surrounding cells and promoting cell-to-cell communication [21–23]. As a related paper, there is a report that miRNA-6780b-5p in exosomes enhances EMT and promotes metastasis [24]. MiR-200 and miR-105 in exosomes secreted from breast cancer induce metastasis [25]. MiR-21-5p and miR-155-5p in macrophage-derived exosomes are transferred to colon cancer and promote cell migration, invasion, and metastasis by inhibiting the expression of BRG1 [26]. Following this research trend, in our study, intercellular communication mediated by exosomal miRNAs was utilized as an important tool to analyze the mechanisms of tumor malignancy.

Our study found that miR-6794-5p was highly expressed in exosomes secreted by Bcl-w-overexpressing U251 and A549 cells. Tumor-derived miR-6794-5p induced tumor malignancy by activating M2 macrophages in the tumor microenvironment and by inducing Interleukin 10 (IL-10) expression. In addition, by analyzing the expression pattern of miR-6794-5p in the plasma of patients and mice, we suggest its potential as a major biomarker for cancer diagnosis and treatment.

## Materials and methods

### Patient specimens

Tissue and plasma from lung cancer patients were Institutional Review Board (IRB) approved in Korea Institute of Radiological and Medical Sciences (KIRAMS). The specimens used for this study were distributed by the Korea Institute of Radiological and Medical Sciences (KIRAMS) Radiation Biobank (KRB) in Republic of Korea (KRB-2021-I002, KRB-2023-I001). The bio-specimens and data used in this study were provided by the Radiation Tissue Resources Bank of Korea Cancer Center Hospital (TB-2021-02-B/P50, C/P50, L/P40). Lung tissue was embedded in paraffin and mRNA extraction and ELISA were performed on plasma.

### Chemicals

Recombinant human IL-10 protein used in the experiment was purchased from R&D Systems (MN, USA). Phorbol 12-myristate 13-acetate (PMA) was purchased from Sigma-Aldrich (MO, USA). Dimethyl amiloride (DMA) was purchased from Santa Cruz Biotechnology (TX, USA).

### Cell culture

LLC1, and WI-38 cells were purchased from American Type Culture Collection (ATCC, VI, USA). U251, A549, and THP-1 cells were purchased from Korean Cell Line Bank (KCLB, Korea). A549 and THP-1 cells were cultured in RPMI 1640 media (Corning, NY, USA). U251 and LLC1 cells were cultured in DMEM (Corning, NY, USA). WI-38 cells were cultured in MEM (Welgene, Korea). All media used for cell culture were supplemented with 10% Fetal Bovine Serum (FBS, Corning, NY, USA) and 1% penicillin streptomycin (Corning, NY, USA). Cells were cultured in an incubator at 37 °C and 5% CO_2_.

### Plasmid, miRNA mimic, and transfection

miR-6794-5p mimic, miR-6794-5p inhibitor, and cy3-tagged miR-6794-5p were synthesized by Genolution Inc. (Korea). All siRNAs against Rab27a, IL-10, SOCS1, and STAT3 purchased from Santa Cruz Biotechnology (TX, USA). The pLPC-flag-SOCS1 vector was provided by Addgene (Plasmid #129514). The Bcl-w gene was inserted into the pcDNA3.1 vector. The following primers were used for Bcl-w overexpression vector; ATGGCGACCCCAGCCTCG (forward) and TCACTTGCTAGCAAAAAAGGCCCCTA (reverse). HindIII and XhoI were used as restriction enzymes. Plasmid, miRNA, and siRNA were transfected into cells using Lipofectamine 2000 reagent (Invitrogen, MA, USA) following the manufacturer’s instructions.

### Transwell-invasion and migration assays

For the invasion assay, a transwell with 8 μm pores (Corning, NY, USA) was coated with matrigel (Corning, NY, USA). The cells were seeded on the matrigel-coated transwell and filled the lower chamber with complete medium. After 16 hours, the transwell was fixed with methanol and stained with crystal violet. For the transwell migration assay, the bottom of the transwell was coated with 0.2% gelatin. Cells were seeded on the matrigel-coated transwell and filled the lower chamber with complete medium. After  16 hours, the transwell was fixed with methanol and stained with crystal violet. For the wound healing assay, 80–90% of the cells are seeded in 12-well plates. Scratch a straight line through the center of the well using a pipette tip. After 16 hours, the number of migrated cells is counted and displayed in a graph.

### Sphere formation assay

The transfected U251, A549, and LLC1 cells were resuspended in Dulbecco’s modified Eagle’s medium-F12 (Gibco, MA, USA) containing B27 (Gibco, MA, USA) and grown for 7–10 days. Spheres were counted with a diameter > 20 μm under an inverted microscope (Olympus, JAPAN).

### Western blot analysis

Proteins were extracted from cells using lysis buffer (10 mM Tris-HCl with pH 7.4, 150 mM NaCl, 1% NP-40, 1 mM EDTA, 0.1% SDS) containing protease inhibitors (Roche, Switzerland) and phosphatase inhibitors (Roche, Switzerland). Protein samples were separated by electrophoresis on SDS-PAGE gel and then transferred to PVDF membranes. The membrane was incubated overnight at 4 °C using the following antibodies; Rab27a (GTX109180) was purchased from Genetex (CA, USA). CD9 (EXOAB-CD9A-1), CD63 (EXOAB-CD63A-1), and TSG101 (EXOAB-TSG101–1) were purchased from System biosciences (CA, USA). Bcl-w (#2724), N-cadherin (#13116), Vimentin (#5741), Zeb1 (#3396), β-catenin (#9582), Notch2 (#5732), Oct4 (#2750), SOCS1(#3950), *p*-Stat3 (#9145), STAT3 (#9139), *p*-JAK1 (#3331), and JAK1 (#3344) were obtained from Cell signaling (MA, USA). Twist (ab50887), CD163 (ab182422), CD206 (ab64693), and CD11b (ab133357) were purchased from Abcam (UK) and β-actin(sc-47,778) was obtained from Santa Cruz Biotechnology (TX, USA). Horseradish peroxidase (HRP) conjugated secondary antibodies (Bio-rad, CA, USA) were incubated for 1 hours in room temperature.

### Total RNA isolation and quantitative real-time PCR

RNA was extracted from cells and plasma using Trizol reagent (Qiagen, Germany). Complementary DNA (cDNA) was synthesized according to the manufacturer’s instructions of the SensiFAST™ cDNA Synthesis Kit (Bioline, OH, USA) and Mir-X™ miRNA First-Strand Synthesis Kit (Takara, JAPAN). Real-time PCR was performed with Power SYBR™ Green PCR Master Mix (Applied Biosystems, MA, USA). The following primers were used for real-time PCR: miR-6794-5p, CAGGGGGACTGGGGGTGAG; Oct4, AGTGAGAGGCAACCT GGAGA (forward) and ACACTCGGACCACATCCTTC (reverse); Zeb1, GCCAATAAGCA AACGATTCTG (forward) and TTTGGCTGGATCACTTTCAAG (reverse); Twist, GAAGAT CATCCCCACGCTG (forward) and AGGAAGTCGATGTACCTGGC (reverse); PRMT1, CC AGTGGAGAAGGTGGACAT (forward) and CTCCCACCAGTGGATCTTGT (reverse); BTG2, CCCTATGAGGTGTCCTACCG (forward) and AGCACTTGGTTCTTGCAGGT (reverse); LZTS1, GAGCCTCATGAAGGAGCAGG (forward) and CAGGTCCTGGGTCCT CAGCT (reverse); SZRD1, AAGTCCCTAGCACAGCGAGA (forward) and GGTTTCTCC TGCTCCTCCTC (reverse); SOCS1, TGGTAGCACACAACCAGGTG (forward) and GAG GAGGAGGAAGAGGAGGA (reverse); CD163, CCAGTCCCAAACACTGTCCT (forward) and CACTCTCTATGCAGGCCACA (reverse); CD206, ACGGACTGGGTTGCTATCAC (forward) and TGATCCCCAAAAGTGTGTCA (reverse); CD11b, ACGTAAATGGGGACA A GCTG (forward) and GATCCTGAGGTTCCGTGAAA (reverse); IL-1b, GGACAAGCTGAG GAAGATGC (forward) and TCGTTATCCCATGTGTCGAA (reverse); IL-10, CCAAGCTG AGAACCAAGACC (forward) and GGGAAGAAATCGATGACAGC (reverse); CCL17, AG CCATTCCCCTTAGAAAGC (forward) and CTGCCCTGCACAGTTACAAA (reverse); CCL22, CGCGTGGTGAAACACTTCTA (forward) and ATCTTCACCCAGGGCACTCT (reverse); CCL24, GCCTTCTGTTCCTTGGTGTC (forward) and GACCACTCGGTTCTCA GGAA (reverse); VEGF, GACAGACAGACAGACACCGCC (forward) and GAACAGCCC AGAAGTTGGACG (reverse); EGF, CCTGGGAATGTGATTGCTTT (forward) and GGCAA ACAGCAAAAATGGTT (reverse); TGFB1, GTGGAAACCCACAACGAAAT (forward) and CGGAGCTCTGATGTGTTGAA (reverse); GAPDH, CATCTCTGCCCCCTCTGCTGA (forward) and GGATGACCTTGCCCACAGCCT (reverse).

### Luciferase reporter assay

The binding site of miR-6794-5p in SOCS1 3’UTR was inserted into the vector, pmirGLO dual-luciferase miRNA target expression vector (Promega, WI, USA). A549 cells, which were seeded in 24well plate, were co-transfected with miR-6794-5p, renilla and luciferase vectors. After 48 hours, luciferase activity was measured using the dual-luciferase reporter assay system (Promega, WI, USA).

### Ago2-RNA immunoprecipitation

This experiment was performed using RIP-assay kit for microRNA (MBL International Corporation, MA, USA) according to the manufacturer’s instructions. Cell lysates were obtained from A549 cells transfected with miR-6794-5p. Agarose beads (Santa Cruz Biotechnology, TX, USA) immobilized with Ago2 antibody (Sigma-Aldrich, MO, USA) were added to the cell lysate to lead immunoprecipitation. RNA is isolated from the formed Ago2 antibody-agarose bead-ribonucleoprotein (RNP) complex, and the RNA level of SOCS1 is measured by real-time PCR.

### Elisa

IL-10 was analyzed in THP-1-derived macrophages and patient plasma using Human IL-10 quantikine ELISA kit (R&D Systems, MN, USA). Debris was removed from the conditioned media of the cells and patient plasma by centrifugation, and then proceeded according to the manufacturer’s instructions. Absorbance is measured at 450 nm using a microplate reader.

### Exosome isolation and identification

Exosomes were isolated from the conditioned media of the cells using ExoQuick-TC (System biosciences, CA, USA). Experiments were performed according to manufacturer’s instructions. The ExoQuick-TC solution was added to the medium from which cell debris was removed by centrifugation and stored overnight at 4 °C. The supernatant was removed by centrifugation, and an exosome pellet was obtained. Analysis of the isolated exosomes was performed using a Hitachi H-7600 Transmission Electron Microscope (Hitachi, JAPAN). SeraMir Exosome RNA Purification Column Kit (System biosciences, CA, USA) was used to extract RNA from exosomes. Western blot analysis samples for detecting the expression of exosome markers were prepared by adding RIPA buffer containing protease inhibitors to exosome pellets.

### In vivo assay

LLC1 cells overexpressing NC or anti-miR-6794-5p were subcutaneously injected into the right flank of 6-week-old Balb/c nude female mice. Cells were injected at 5 × 10^5^ cells per mice. Based on the day of injection, they were sacrificed 2 weeks later. The tumor volume was measured width and length and calculated as (width × width × length)/2. LLC1 cells overexpressing NC, miR-6794-5p, or miR-6794-5p + SOCS1 were injected into the tail vein of 6-week-old C57BL/6 female mice. Cells were injected at 2 × 10^5^ cells per mice. Based on the day of injection, they were sacrificed 3 weeks later. Lung tissues were fixed with formaldehyde and made into paraffin blocks. LLC1 cells overexpressing NC or miR-6794-5p mimics were subcutaneously injected into the right flank of 6-week-old C5BL/6 female mice. Cells were injected at 2 × 10^5^ cells per mice. Based on the day of injection, they were sacrificed 2 weeks later. The tumor tissues were analyzed by flow cytometry. RNA was extracted from plasma separated from blood. These studies were reviewed and approved by the Institutional Animal Care and Use Committee (IACUC) of Korea Institute of Radiological & Medical Science.

### Hematoxylin and eosin (H&E) and immunohistochemistry (IHC) staining

Lung tissue fixed in 4% formaldehyde was embedded in paraffin. Paraffin sections are deparaffinized and hydrated. For H&E staining, paraffin sections were stained with Hematoxylin (Dako, CA, USA) and eosin (Epredia, NH, USA). For IHC, paraffin sections were subjected to Target retrieval solution (Dako, CA, USA), and endogenous peroxidase was blocked using H_2_O_2_. Antibodies used in IHC were CD206 (Abcam, UK), SOCS1 (Genetex, CA, USA). After reacting with biotinylated secondary antibody, DAB staining is performed. The signal was detected using cellSens (Olympus, JAPAN).

### Fluorescence image analysis

Cells were transfected with cy3-tagged miR-6794-5p (Genolution, KOREA), cultured, and fixed with 4% paraformaldehyde solution. The cell morphology was confirmed by brightfield, and the fluorescence of cy3 expressed in the cells was measured using an INCELL2000 analyzer (GE Healthcare, IL, USA).

### Flow cytometry

For flow cytometry, tumor tissues harvested from mice were prepared using a Tumor dissociation kit (Miltenyi biotec, Germany) according to the manufacturer’s instructions. All antibodies used in the flow cytometer were purchased from Biolegend (CA, USA). Single cells were stained with the following antibody; Zombie NIR™ fixable viability kit (423105), FITC anti-mouse I-A/I-E antibody (107605), PE anti-mouse F4/80 antibody (123109), PE/Cyanine7 anti-mouse/human CD11b antibody (101215), APC anti-mouse CD206 (MMR) antibody (141707), and Brilliant violet 510™ anti-mouse CD45 antibody (103137). Samples were acquired with a CytoFLEX flow cytometer (Beckman Coulter, CA, USA).

### MiRNA microarray analysis

MiRNA microarray analysis was performed using exosomes isolated from the conditioned media of U251 cells overexpressing control vector and Bcl-w. MiRNA microarray analysis was provided by MACROGEN (Korea). The Affymetrix Genechip miRNA array process was performed according to the manufacturer’s protocol. Comparative analysis between test and control samples was performed using fold change all statistical tests. Visualization of differentially expressed genes was performed using the R statistical language v. 2.15.0.

## Statistical analysis

GraphPad software was used for all data analysis. All data are expressed as mean ± SD. Statistical calculations were performed with Student’s t-test or One-way ANOVA followed by bonferroni comparison test.

## Results

### Secretion of exosomes is involved in Bcl-w-induced tumorigenicity

Our previous study showed that the overexpression of Bcl-w in glioblastoma multiforme (GBM) cells promotes tumor aggressiveness [9]. To analyze the cause of Bcl-w-induced cancer malignancy, exosomes were selected as the connection medium between tumor cells and the surrounding microenvironment, and the effects of exosomes secreted from cancer cells on the tumor microenvironment were assessed. First, we attempted to confirm whether exosome secretion affected Bcl-w-induced cancer malignancy. Rab27a is a member of the Rab family of small GTPases and is involved in exosome secretion [27, 28]. Dimethyl amiloride (DMA) inhibits exosome release by interfering with intracellular calcium channel mechanisms [29, 30]. The use of Rab27a siRNA or DMA to inhibit exosome secretion reduced Bcl-w-induced migratory ability, invasiveness, and stemness maintenance (Fig. S1A-E). These results suggest that exosomes are involved in Bcl-w-induced tumorigenicity.

Numerous factors such as proteins, DNA, RNA, and lipids are contained in exosomes [20]; however, in this study, we focused on miRNAs involved in the expression of oncogenic factors. In particular, exosomal miRNAs regulate various biological phenomena such as metastasis, treatment resistance, tumor growth, epithelial–mesenchymal transition (EMT), and angiogenesis, and are involved in cell-to-cell communication within the tumor microenvironment [31, 32]. Therefore, exosomes isolated from the conditioned media of the control vector or Bcl-w-overexpressing GBM U251 cells were compared using a miRNA microarray. To confirm the isolation of exosomes, the size of the isolated vesicles was confirmed using a transmission electron microscope (TEM) (Fig. S2A). In addition, it was confirmed that the expression of the exosome markers CD9, CD63, and TSG101 was higher in the conditioned media obtained from Bcl-w overexpressing cells than in the control vector cells (Fig. S2B).

### Bcl-w-induced exosomal miR-6794-5p promotes tumor progression

**Fig. 1:**
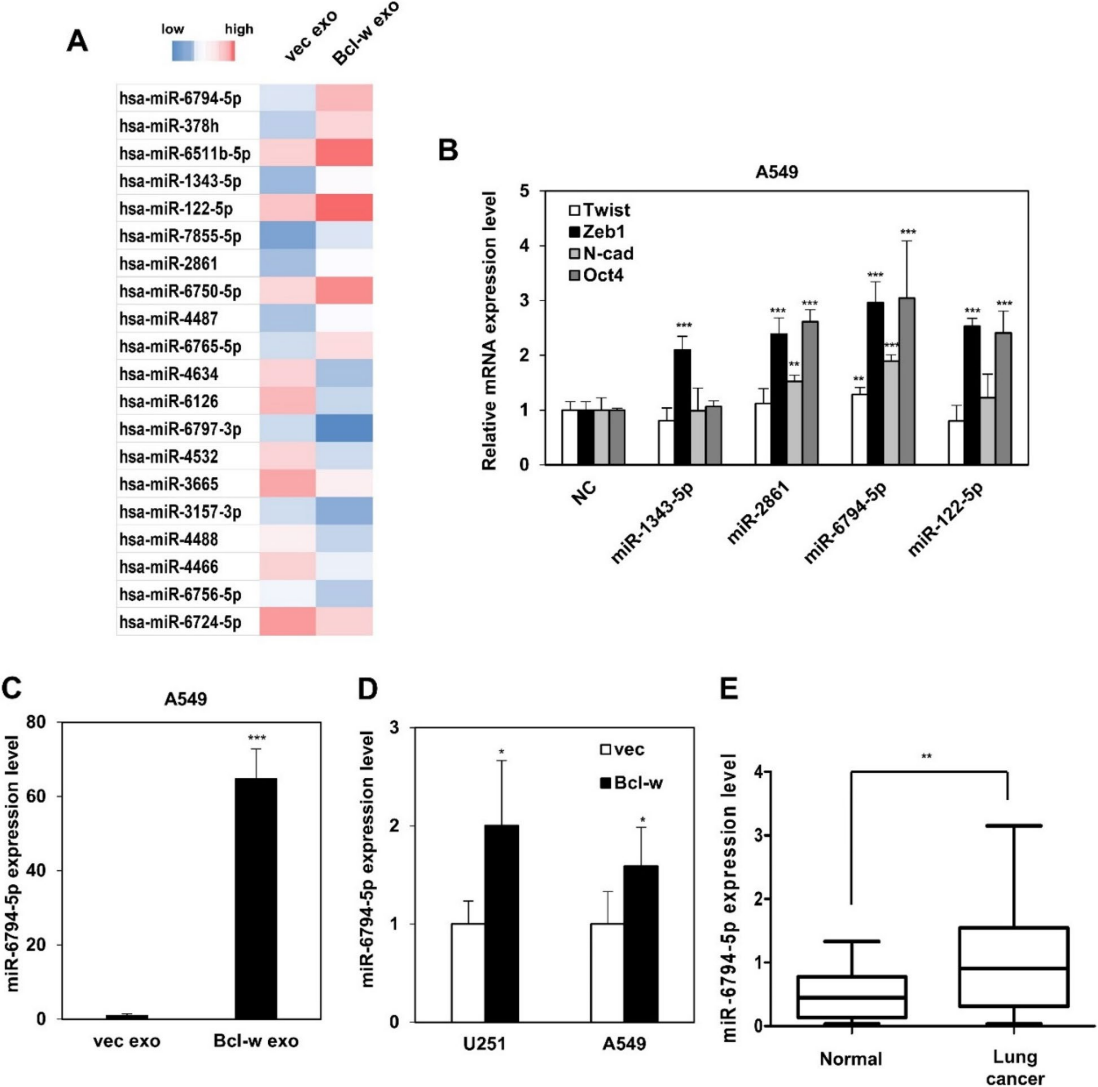
MiR-6794-5p is upregulated within Bcl-w-derived exosomes. **A** Heatmap showing expressed exosomal miRNAs between vector and Bcl-w. **B** A549 cells were transfected with the negative control (NC), miR-1343-5p, miR-2861, miR-6794-5p, and miR-122-5p mimics, respectively. The mRNA expression levels of Twist, Zeb1, N-cad, and Oct4 by each miRNA were measured by qRT-PCR. The values were normalized to GAPDH. **C** Expression level of miR-6794-5p in exosomes isolated from conditioned media from Bcl-w-overexpressing A549 cells. **D** miR-6794-5p expression levels in Bcl-w overexpressing U251 and A549 cells. **E** The expression of miR-6794-5p in plasma of normal and patients with lung cancer (normal, *n* = 29; lung cancer, *n* = 24) was analyzed by qRT-PCR. The values were normalized to U6. Data are presented as mean ± SD after triplicate. *P< 0.05; **P<0.01; ***P<0.001. Student's t-test

**Fig. 2:**
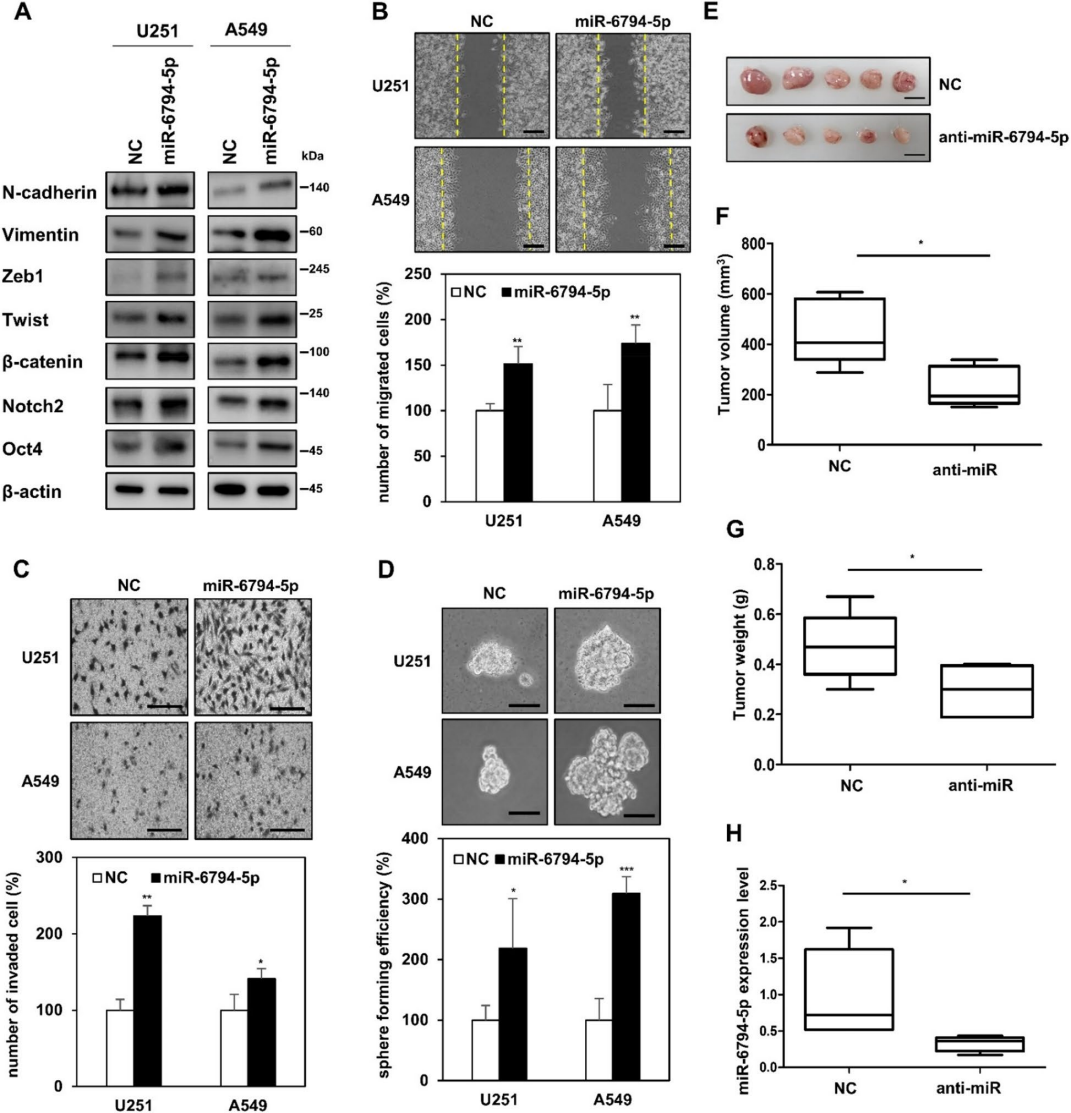
miR-6794-5p promotes tumorigenicity. **A**-**D** U251 and A549 cells were transfected with either negative control (NC) or miR-6794-5p mimic. **A** Expression of mesenchymal and stemness marker was confirmed with Western blot analysis in indicated cells. β-actin was used for normalization. The experiment was repeated with triplicates and representative Western blotting images are shown. Wound healing (**B**), matrigel coated invasion (**C**), and sphere forming (**D**) assays were subjected on the indicated cells. Scale bar is 200 μm. **E**-**H** LLC1 cells overexpressing negative control (NC) or miR-6794-5p inhibitor (anti-miR-6794-5p) were subcutaneously injected into the right flank of Balb/c nude mice (*n* = 5; 5 X 10^5^ cell/mice), respectively. After harvesting mice on 14 days, whole tumor images (**E**), tumor volumes (**F**), and tumor weights (**G**) obtained from the negative control (NC) and anti-miR-6794-5p overexpressing groups. **H** The expression of miR-6794-5p in tumor tissue of negative control (NC) and anti-miR-6794-5p overexpressing groups was analyzed by qRT-PCR. The data are presented as the mean ± S.D. after triplicate. *P < 0.05; **P < 0.01; ***P < 0.001. Student’s t-test

As a result of the miRNA microarray, a total of 20 miRNAs were displayed on the heat map compared to exosomes isolated from control vector (vec exo) versus Bcl-w overexpressing cancer cells (Bcl-w exo). In Bcl-w exo, 10 of these miRNAs were up-regulated and the remaining 10 were down-regulated (Fig. 1A, Table 1). Among the top onco-miRNAs, we selected four candidate miRNAs: miR-1343-5p, miR-2861, miR-6794-5p, and miR-122-5p. Comparison of the mRNA expression levels of Twist, Zeb1, N-cadherin, and Oct4 in A549 cells overexpressing each of the four candidate miRNA mimics showed that miR-6794-5p resulted in the greatest increase (Fig. 1B). To confirm the selected results from the array, we showed that the expression of miR-6794-5p was increased in exosomes isolated from conditioned media from Bcl-w overexpressing U251 and A549 cells (Fig. 1C, Fig. S3). Expression of miR-6794-5p was also increased in U251 and A549 Bcl-w overexpressing cells (Fig. 1D). In addition, the expression of miR-6794-5p was increased in the plasma of patients with lung cancer compared to that in the normal group, showing its potential as an onco-miRNA (Fig. 1E). To analyze the mechanism of miR-6794-5p, we transfected miR-6794-5p mimic into U251 and A549 cells. Overexpression of miR-6794-5p mimics improved the protein expression levels of EMT- and stemness-related markers (Fig. 2A) as well as cell migration (Fig. 2B), invasion (Fig. 2C), and stemness maintenance (Fig. 2D). Exosomes derived from U251 cells overexpressing miR-6794-5p were isolated to confirm the results shown in Fig. 2 (Fig. S4A). First, we confirmed that the expression of miR-6794-5p in isolated exosomes was increased (Fig. S4B). Exosomes overexpressing miR-6794-5p increased EMT and tumor stem cell-related protein expression (Fig. S4C), cell motility (Fig. S4D), invasiveness (Fig. S4E), and stemness maintenance (Fig. S4F). These results suggest that the overexpression of miR-6794-5p in exosomes strongly promotes cancer malignancy by inducing EMT, migration, invasion and stemness in cancer cells. To confirm the role of miR-6794-5p in vivo, we overexpressed miR-6794-5p in a mouse Lewis lung cancer cell line (LLC1) and confirmed its phenotype. The protein expression of EMT and stemness-related markers (Fig. S5A), cell motility (Fig. S5B), invasive ability (Fig. S5C), and stemness (Fig. S5D) were enhanced by miR-6794-5p. Subcutaneous injection of LLC1 cells overexpressing anti-miR-6794-5p decreased tumor volume and weight (Fig. 2E-G) as well as the level of miR-6794-5p expression in mouse tumor tissues (Fig. 2H). Conversely, overexpression of the miR-6794-5p mimic increased the tumor volume, tumor weight (Fig. S6A-C), and miR-6794-5p expression in the plasma of the mice (Fig. S6D).

**Table 1:** Expression levels of miRNAs in exosomes isolated from the conditioned media  of U251 cells overexpressing vector and Bcl-w

Exosomal miRNAs	Fold change values
hsa-miR-6794-5p	3.036649
hsa-miR-378 h	2.99539
hsa-miR-6511b-5p	2.882848
hsa-miR-1343-5p	2.818401
hsa-miR-122-5p	2.777872
hsa-miR-7855-5p	2.742615
hsa-miR-2861	2.514462
hsa-miR-6750-5p	2.338824
hsa-miR-4487	2.325015
hsa-miR-6765-5p	2.300397
hsa-miR-4634	− 3.93943
hsa-miR-6126	−3.76871
hsa-miR-6797-3p	−3.42314
hsa-miR-4532	−2.51446
hsa-miR-3665	−2.25797
hsa-miR-3157-3p	−2.09922
hsa-miR-4488	−2.09922
hsa-miR-4466	−1.92637
hsa-miR-6756-5p	−1.88566
hsa-miR-6724-5p	−1.8684

### miR-6794-5p enhances tumorigenicity by down-regulating SOCS1

**Fig. 3:**
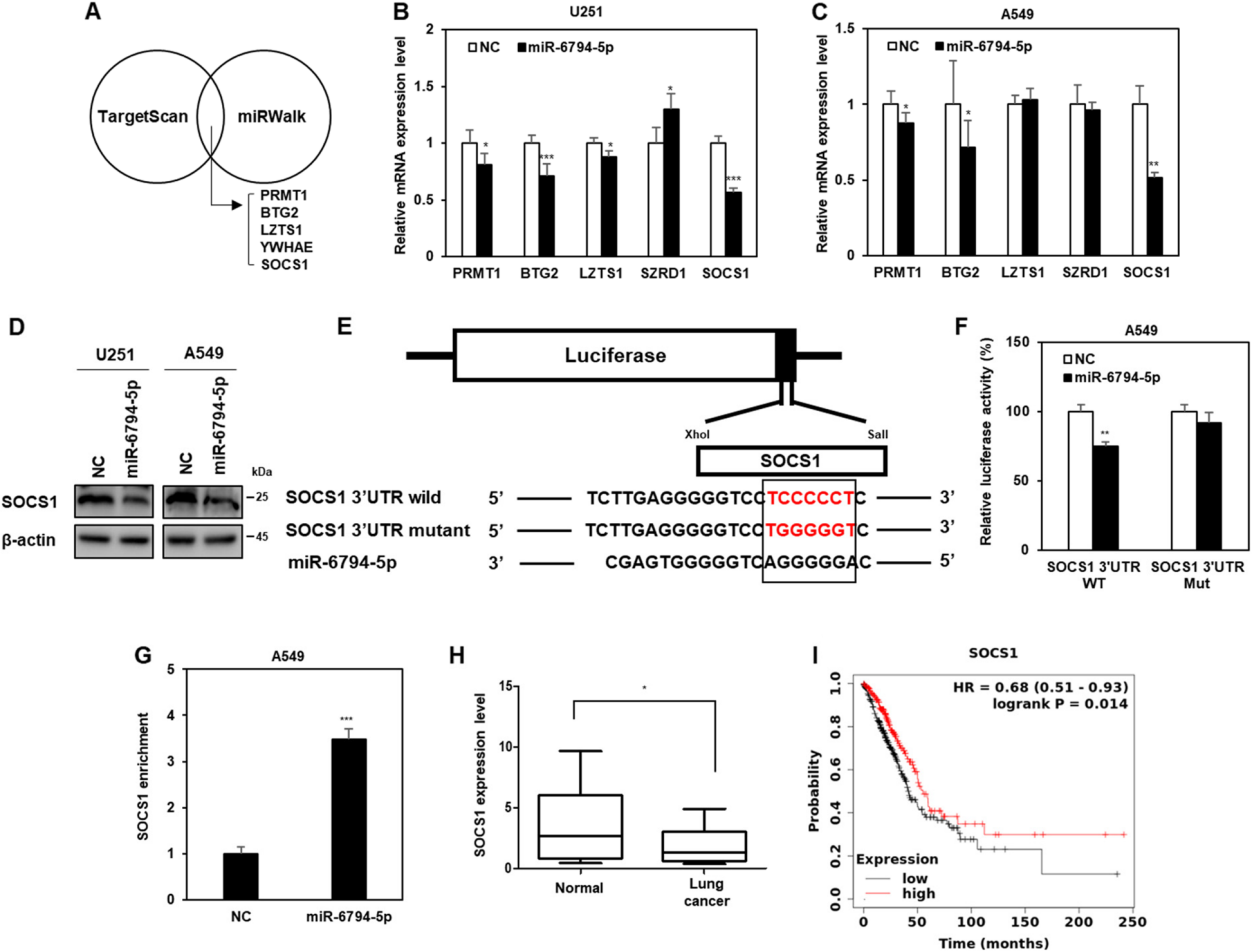
miR-6794-5p directly inhibits the expression of SOCS1. **A** Venn diagram indicated target candidate genes of miR-6794-5p using TargetScan and miRWalk, which are miRNA target prediction sites. **B, C** After overexpression of the miR-6794-5p mimic in U251 (**B**) and A549 (**C**) cells, the mRNA expression of each of the candidate genes was confirmed by qRT-PCR. The values were normalized to GAPDH. **D** After miR-6794-5p was overexpressed in both cells, the level of SOCS1 protein was confirmed by Western blot analysis. β-actin was used for normalization. The experiment was repeated with triplicates and representative Western blotting images are shown. **E, F** Dual luciferase activity was examined after A549 cells were co-transfected with wild-type (WT) or mutant (Mut) vectors of the SOCS1 3’UTR in the presence or absence of the miR-6794-5p mimic, respectively. **G** Ago2-RNA immunoprecipitation (Ago2-IP) assay was performed in negative control (NC) or miR-6794-5p overexpressed A549 cells, and SOCS1 enrichment was confirmed by qRT-PCR. The values were normalized to GAPDH. **H** The mRNA expression of SOCS1 in plasma of normal and patients with lung cancer (normal, *n* = 23; lung cancer, *n* = 23) was analyzed by qRT-PCR. **I** Kaplan-Meier plots were used to compare survival rates between normal groups and lung adenocarcinoma patients. All data are presented as the mean ± S.D. after triplicate. *P < 0.05; **P < 0.01; ***P < 0.001. Student’s t-test

**Fig. 4:**
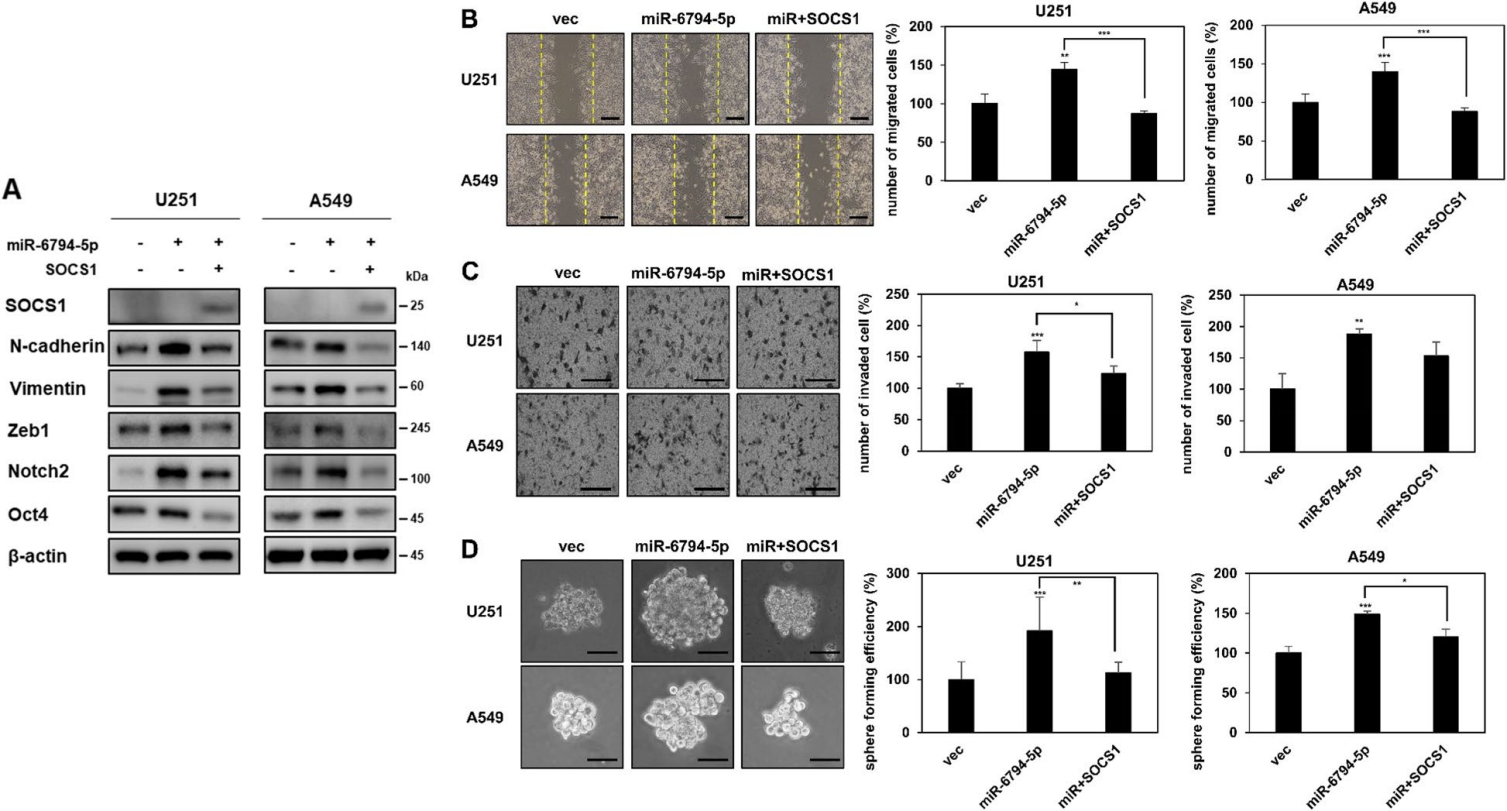
miR-6794-5p promotes EMT, cell mobility, invasiveness, and stemness maintenance by suppressing SOCS1. **A**-**D** U251 and A549 cells were co-transfected with miR-6794-5p or SOCS1 overexpressing vectors. **A** The expression of EMT and stemness marker proteins in the indicated cells was confirmed by Western blot analysis. β-actin was used for normalization. The experiment was repeated with triplicates and representative Western blotting images are shown. Wound healing (**B**), matrigel coating invasion (**C**), and sphere formation assays (**D**) were performed to confirm cell mobility, invasiveness, and stemness maintenance in the indicated cells. Scale bar is 200 μm. The data are presented as the mean ± S.D. after triplicate. *P < 0.05; **P < 0.01; ***P < 0.001. One-way ANOVA followed by bonferroni comparison test

To identify the target genes of miR-6794-5p, we used TargetScan and miRWalk, tools for predicting the target genes of miRNAs [33, 34] (Fig. 3A). We selected PRMT1, BTG2, LZTS1, SZRD1, and SOCS1 as candidate target genes for direct binding of miR-6794-5p. When miR-6794-5p was overexpressed in U251 and A549 cells, SOCS1 mRNA and protein expression were dramatically suppressed (Fig. 3B-D). To confirm that miR-6794-5p directly binds to the SOCS1 gene and regulates its expression, luciferase activity assays were performed using wild-type or mutant constructs from the 3’UTR of SOCS1 (Fig. 3E). When miR-6794-5p and the wild-type of SOCS1 3’UTR were co-transfected, the luciferase activity was reduced compared to that in the control group, whereas when miR-6794-5p and mutant type of the SOCS1 3’UTR were co-transfected, the activity was not significantly different from control group (Fig. 3F). Since the Ago2 protein and miRNA complex binds to the target factor and inhibits the translation of its mRNA, we sought to verify that it is a direct target by confirming the binding between miR-6794-5p and SOCS1 through Ago2-RNA immunoprecipitation analysis. Upon overexpression of miR-6794-5p, the binding of Ago2 protein and SOCS1 mRNA was increased (Fig. 3G). These results showed that miR-6794-5p binds to Ago2 protein and directly reduces target SOCS1 expression. The purpose of this study was to compare the expression of SOCS1 in plasma samples from normal participants and patients with lung cancer and to evaluate its association with survival. As a result, we confirmed that the mRNA expression of SOCS1 was decreased in plasma of patients with lung cancer compared to that in the normal group (Fig. 3H). Additionally, the survival curves of patients with lung cancer using Kaplan-Meier (KM) plot analysis showed that low SOCS1 expression was associated with poor prognosis (Fig. 3I). Analysis of The Cancer Genome Atlas Program (TCGA) dataset revealed that patients with stage IV lung adenocarcinoma had lower SOCS1 expression than those with stage I (Fig. S7). The effects of miR-6794-5p and its target gene SOCS1 on the mechanism of tumorigenicity were analyzed. The miR-6794-5p mimic induced the expression of EMT- and stemness-related factors (N-cadherin, Vimentin, Zeb1, Notch2, and Oct4) (Fig. 4A), migratory ability (Fig. 4B), invasiveness (Fig. 4C), and sphere formation ability (Fig. 4D) were reduced by SOCS1 overexpression.

### Tumor-derived miR-6794-5p enhances tumorigenicity through interaction with macrophages

Cancer cells are surrounded by a tumor microenvironment composed of extracellular matrix, immune cells, endothelial cells, and fibroblasts. The delivery of exosomes into the tumor microenvironment is known to be involved in tumor initiation, progression, angiogenesis, and metastasis [35, 36]. To select stromal cells involved in cancer malignancy among the components of the tumor microenvironment, THP-1-derived macrophages or lung fibroblasts WI-38 were co-cultured with both cancer cells, respectively (Fig. S8). Cell migratory ability and invasiveness were improved in both cancer cell lines treated with conditioned media obtained from THP-1-derived macrophages overexpressing miR-6794-5p, compared to that of negative control (Fig. S8A-C). In contrast, there was no significant difference in EMT- or stemness-related protein expression, cell mobility, or invasiveness of U251 and A549 cells treated with conditioned media obtained from miR-6794-5p-overexpressing fibroblasts, WI-38, between the two groups (Fig. S8D-F). Based on these results, we hypothesized that some factors present in the conditioned media obtained from THP-1-derived macrophages overexpressing miR-6794-5p are involved in cancer malignancy.

### IL-10 secretion induced by miR-6794-5p in macrophages promotes cancer malignancy

**Fig. 5:**
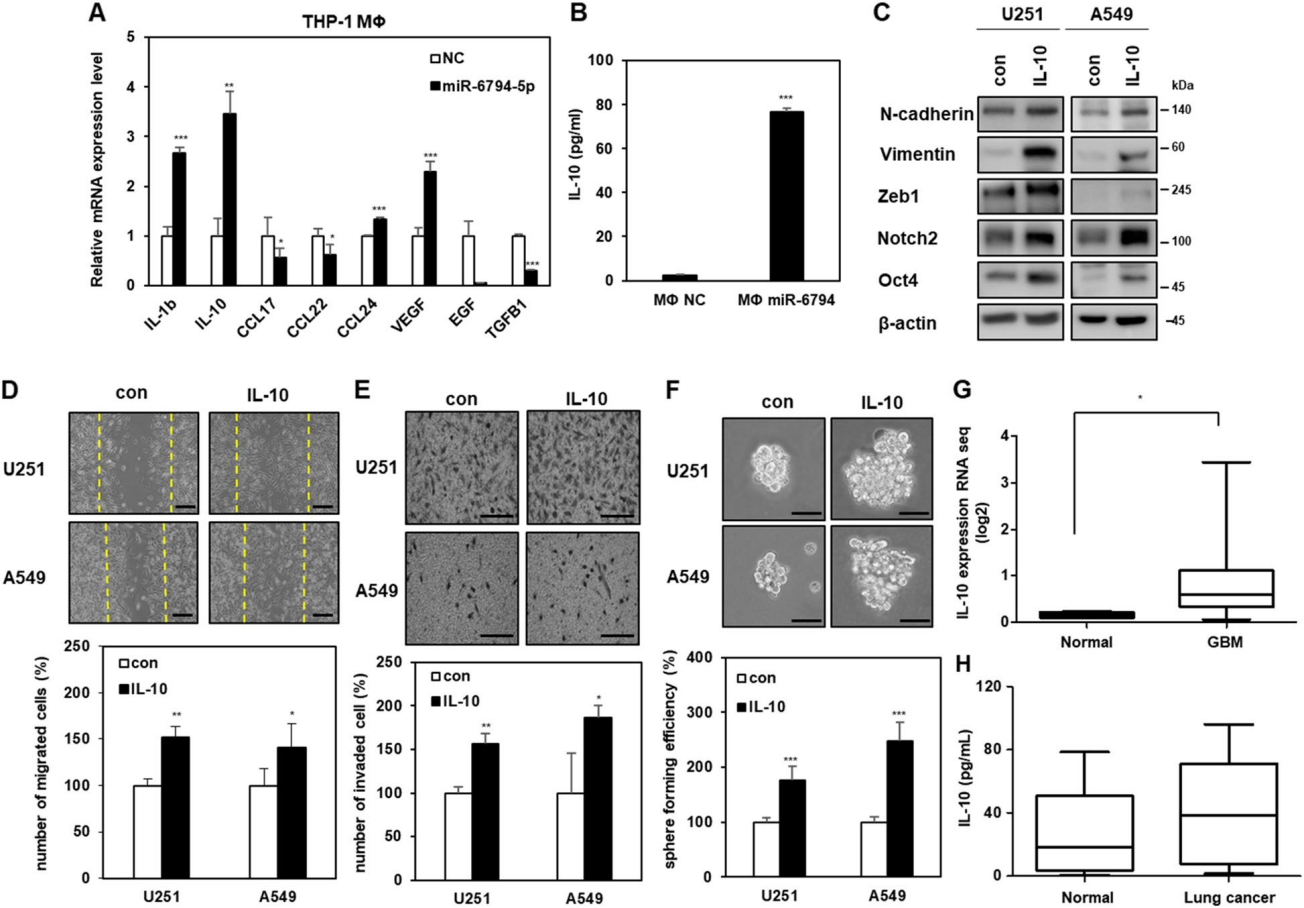
miR-6794-5p-induced IL-10 in THP-1-derived macrophages contributes to tumorigenicity. **A** mRNA expression levels of cytokines and growth factors in miR-6794-5p overexpressed THP-1-derived macrophages were examined by qRT-PCR. The values were normalized to GAPDH. **B** Expression of IL-10 was reconfirmed in the conditioned media of miR-6794-5p overexpressed THP-1 derived macrophages using ELISA. **C**-**F** After treatment with recombinant IL-10 (5 ng/ml) on U251 and A549, Western blot (**C**), wound healing (**D**), matrigel coated invasion (**E**), and sphere formation assays (**F**). β-actin was used for normalization in Western blot analysis. The experiment was repeated with triplicates and representative Western blotting images are shown. Scale bar is 200 μm. **G** The expression of IL-10 was shown in box and whisker plots by comparing normal (*n* = 5) and GBM patients (*n* = 168) from TCGA GBM cohort within the Genomic Data Common (GDC). **H** The expression of IL-10 in the plasma of normal (*n* = 22) and patients with lung cancer (*n* = 19) was assayed by ELISA. Data are presented as mean ± SD after triplicate. *P < 0.05; **P < 0.01; ***P < 0.001. Student’s t-test

We measured the mRNA expression of malignant cancer factors secreted by macrophages overexpressing miR-6794-5p using qRT-PCR. Among the secreted factors identified, IL-1β and IL-10 are anti-inflammatory cytokines [37, 38], and CCL17 and CCL24 are known chemokines that attract immunosuppressive cells [39, 40]. Additionally, vascular endothelial growth factor (VEGF) and epidermal Growth Factor (EGF) have been reported to promote angiogenesis and cancer cell progression [41, 42]. IL-10 mRNA expression was most dramatically increased in miR-6794-5p-overexpressing THP-1-derived macrophages (Fig. 5A). In the conditioned media of miR-6794-5p-overexpressing THP-1-derived macrophages, the secretion of IL-10 was increased compared to that in the negative control group (Fig. 5B). To examine the effect of the increased IL-10 on the surrounding cancer cells, recombinant IL-10 was overexpressed in U251 and A549 cells, and the malignant phenotype was confirmed in both cell lines. Overexpression of IL-10 increased the protein expression of N-cadherin, Vimentin, Zeb1, Notch2, and Oct4 as well as cell motility, invasiveness, and sphere formation ability (Fig. 5C-F). RNA sequencing analysis of samples from TCGA patients with glioblastoma (GBM) [43] revealed that IL-10 expression was higher patients with GBM than in normal participants (Fig. 5G). Additionally, IL-10 expression in the plasma of patients with lung cancer was higher than that in normal participants (Fig. 5H). These results suggest that miR-6794-5p contributes to cancer cell malignancy by inducing IL-10 expression in macrophages.

**Fig. 6:**
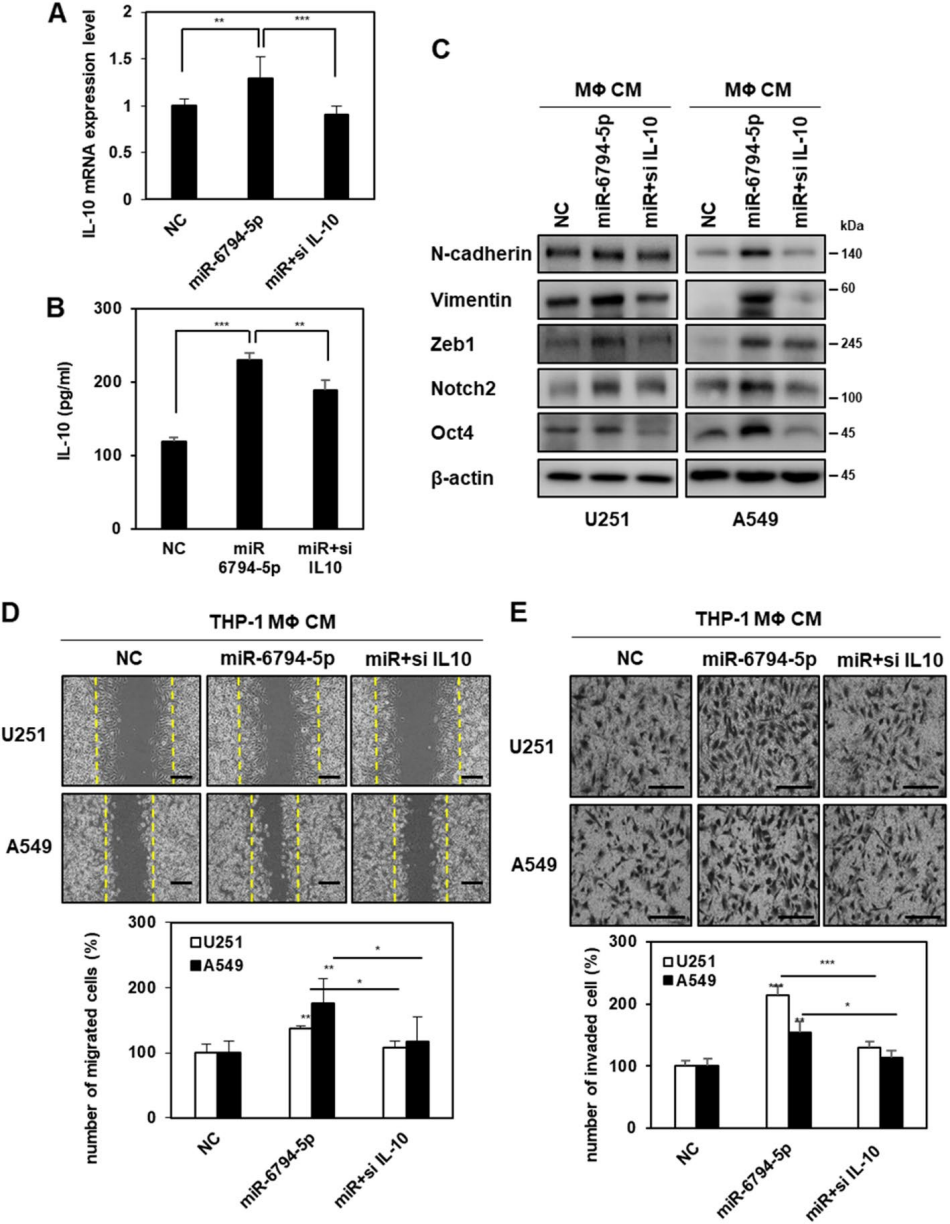
Targeting of IL-10 in M2 macrophages prevents tumor malignancy. **A**-**E** After THP-1 derived macrophages were transfected with miR-6794-5p mimics or siRNA against IL-10 (si IL-10), the indicated cells were subjected to qRT-PCR (**A**), ELISA (**B**), Western blot (**C**), wound healing (**D**), and matrigel coated invasion (**E**) assays. Scale bar is 200 μm. The values were normalized to GAPDH in qRT-PCR. β-actin was used for normalization in Western blot analysis. The experiment was repeated with triplicates and representative Western blotting images are shown. The data are presented as the mean ± S.D. after triplicate. *P < 0.05; ** *P* < 0.01; ***P < 0.001. One-way ANOVA followed by bonferroni comparison test

To further validate the association between miR-6794-5p-induced IL-10 expression and tumor malignancy, THP-1-derived macrophages were co-transfected with miR-6794-5p and siRNA against IL-10. It was confirmed that the mRNA expression of miR-6794-5p-induced IL-10 was reduced by IL-10 knockdown in THP-1-derived macrophages (Fig. 6A). The miR-6794-5p increased IL-10 secretion was also reduced by IL-10 knockdown in the conditioned media of macrophages (Fig. 6B). When U251 and A549 cells were treated with conditioned media from miR-6794-5p-overexpressing macrophages, miR-6794-5p increased the expression of N-cadherin, Vimentin, Zeb1, Notch2, and Oct4 (Fig. 6C), cell migration (Fig. 6D), and invasiveness (Fig. 6E) were reduced by IL-10 knockdown.

### miR-6794-5p promotes M2 polarization by activating the JAK1/STAT3 pathway in macrophages

**Fig. 7:**
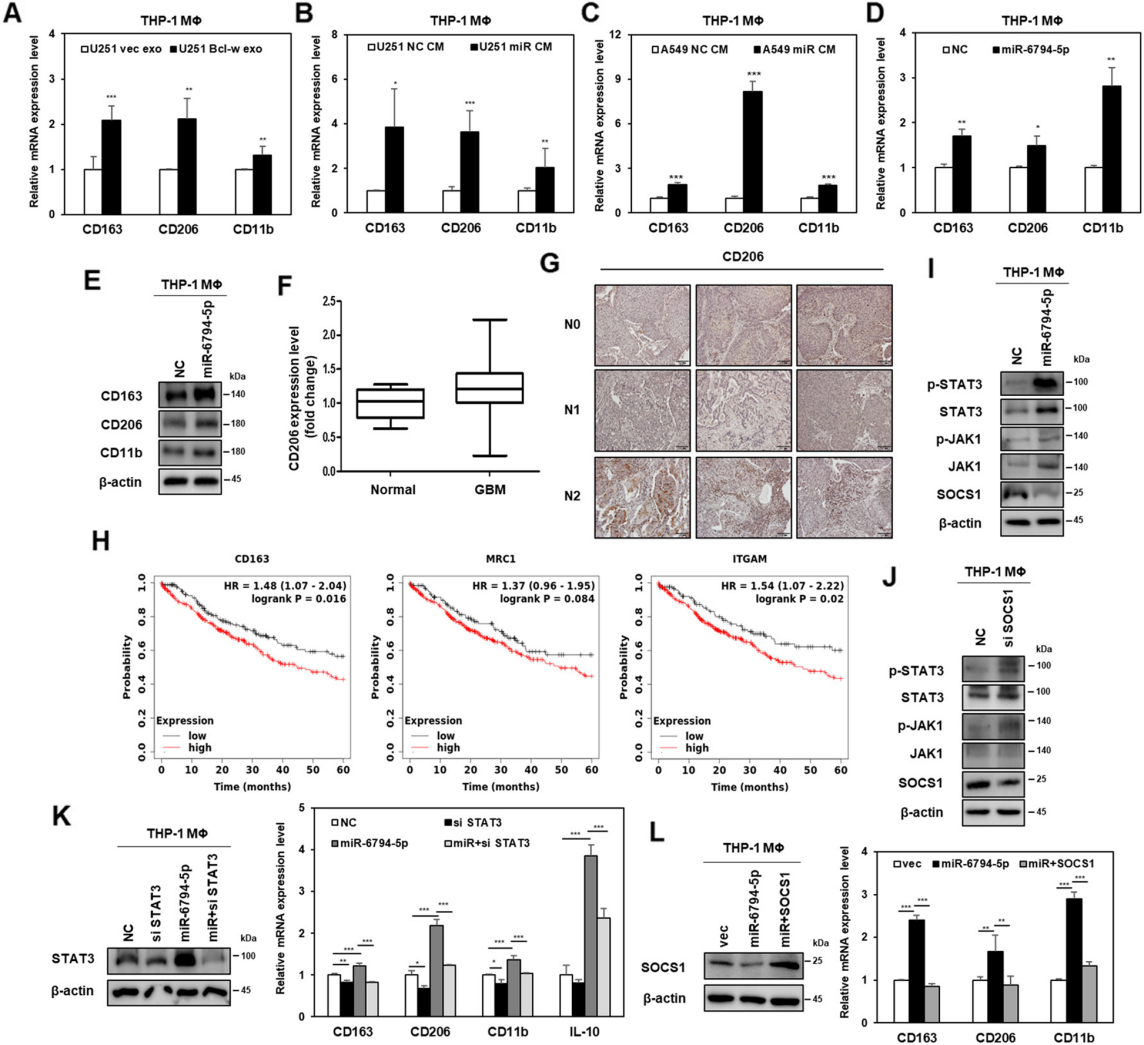
miR-6794-5p induces macrophage M2 polarization via JAK1/STAT3 pathway. **A** Exosomes were isolated from the conditioned media of U251 cells overexpressing Bcl-w. After treatment of THP-1-derived macrophages with exosomes, the mRNA levels of CD163, CD206, and CD11b were measured by qRT-PCR analysis**. B, C** After THP-1-derived macrophages were treated with conditioned media from U251 and A549 cells transfected with miR-6794-5p mimics, respectively, mRNA levels of macrophage M2 markers (CD163, CD206, and CD11b) were measured by qRT-PCR analysis. **D, E** mRNA (**D**) or protein (**E**) levels of macrophage M2 markers (CD163, CD206, and CD11b) in THP-1-derived macrophages overexpressing the miR-6794-5p mimic were verified by qRT-PCR or Western blot analysis, respectively. **F** Using the TCGA dataset, expression of CD206 was compared with normal (*n* = 5) and GBM patients (*n* = 167) and displayed as box and whisker plots. **G** The expression level of CD206 in the lung tissues at each stage of lymph node metastasis of patients with lung cancer was confirmed by IHC. **H** Kaplan-Meier overall survival curves of patients with lung squamous cell carcinoma according to the expression of CD163, CD206 (MRC1), and CD11b (ITGAM). **I, J** Expressions of p-STAT3, STAT3, p-JAK1, JAK1, and SOCS1 in miR-6794-5p-overexpressed (**I**) or SOCS1-knockdown (**J**) THP-1-derived macrophages were shown by Western blot analysis. **K** Expression of STAT3 protein after transfection with STAT3 against siRNA in macrophages overexpressing miR-6794-5p (left). Expression mRNA levels of macrophage M2 markers (CD163, CD206, and CD11b) and IL-10 were checked in the indicated cells (right). **L** After overexpressing empty vector, miR-6794-5p and miR-6794-5p + SOCS1 in THP-1-derived macrophages, the expression of SOCS1 protein was confirmed by Western blot analysis (left), and the mRNA expression levels of macrophage M2 markers (CD163, CD206, and CD11b) were confirmed by qRT-PCR (right) in indicated cells. β-actin was used for normalization in Western blot analysis. The values were normalized to GAPDH in qRT-PCR. The experiment was repeated with triplicates and representative Western blotting images are shown. The data are presented as the mean ± S.D. after triplicate. *P < 0.05; **P < 0.01; ***P < 0.001. **A**-**D** Student’s t-test. **K**,** L** One-way ANOVA followed by bonferroni comparison test

M2 macrophages contribute to accelerate cell proliferation, angiogenesis, tissue repair, and metastasis through various anti-inflammatory mechanisms [44, 45]. Therefore, in this study, we investigated whether exosomal miR-6794-5p, delivered from cancer cells to macrophages, induces M2 macrophage polarization. In addition, we attempted to confirm that exosomes isolated from the conditioned media of cancer cells overexpressing Bcl-w were involved in M2 polarization by directly treating macrophages. When THP-1-derived macrophages were directly treated with Bcl-w-derived exosomes, the mRNA expression of macrophage M2 markers CD163, CD206, and CD11b was upregulated (Fig. 7A). These results supported the feasibility of pathological exosome-based delivery. To confirm whether tumor-derived miR-6794-5p was delivered to the macrophages, THP-1-derived macrophages were treated with exosomes isolated from the conditioned media of cy3-tagged miR-6794-5p-overexpressing U251 cells. Cy3 fluorescence of miR-6794-5p was observed using INCELL2000 analyzer (Fig. S9A, B) and miR-6794-5p RNA levels were measured in the macrophages (Fig. S9C, D). When macrophages with miR-6794-5p-overexpressing exosomes, miR-6794-5p expression increased in macrophages. These results confirmed that tumor-derived miR-6794-5p was delivered to macrophages (Fig. S9B). In macrophages treated with miR-6794-5p-overexpressing U251 and A549 conditioned media, the expression of miR-6794-5p increased (Fig. S9C, D), but the mRNA level of the target gene SOCS1 decreased (Fig. S9E, F) compared to the negative control. In addition, the IL-10 mRNA expression increased compared to that in macrophages treated with the control-conditioned media of U251 and A549 cells (Fig. S9G, H).

When THP-1-derived macrophages were treated with conditioned media from U251 and A549 cells overexpressing miR-6794-5p, the mRNA levels of M2 macrophage markers, such as CD163, CD206 and CD11b, increased. These results suggested that exosomal miR-6794-5p promotes M2 macrophage polarization (Fig. 7B, C). To understand the molecular mechanism underlying the M2 macrophage polarization induced by miR-6794-5p, miR-6794-5p was overexpressed in THP-1-derived macrophages. The mRNA and protein levels of macrophage M2 markers CD163, CD206, and CD11b were increased compared to those in the negative control (Fig. 7D, E). Using TCGA dataset, CD206 expression was found to be higher in patients with GBM than normal participants (Fig. 7F). The lung tissues of patients with lung cancer with lymph node metastasis were classified into stages N0, N1, and N2. As lymph node metastasis progressed, the expression of CD206 in lung tissue increased (Fig. 7G). Analysis of the correlation between Bcl-w and CD206 using TCGA dataset revealed that, the expression of CD206 in glioma and glioblastoma tissues was higher when Bcl-w was expressed at a higher level (Fig. S10). In addition, higher expression of CD163, CD206 (MRC1), and CD11b (ITGAM) in patients with lung squamous cell carcinoma was associated with lower overall survival (Fig. 7H).

STAT3 activation is involved in macrophage M2 polarization [46]. Therefore, we confirmed that the JAK1/STAT3 pathway is involved in the M2 polarization of macrophages by miR-6794-5p. Overexpression of miR-6794-5p in macrophages activates STAT3 and JAK1 and reduces the expression of the target gene, SOCS1 (Fig. 7I). It has been reported that SOCS1 is known to block the JAK/STAT pathway, inhibiting tyrosine phosphorylation by directly binding to the SH2 domains of JAK and STAT [47, 48]. By examining whether miR-6794-5p activates the JAK1/STAT3 pathway by suppressing SOCS1 expression, we confirmed that silencing SOCS1 activates the JAK1/STAT3 pathway (Fig. 7J). To confirm whether miR-6794-5p induces the M2 polarization of macrophages through STAT3, miR-6794-5-overexpressing macrophages were transfected with siRNA against STAT3. siRNA-mediated STAT3 knockdown reduced miR-6794-5p-induced expression of M2 macrophage markers (CD163, CD206, and CD11b) and IL-10 (Fig. 7K). The effects of miR-6794-5p and its target SOCS1, on M2 macrophage polarization were analyzed. miR-6794-5p-induced M2 marker levels were reduced by the overexpression of SOCS1 (Fig. 7L). These results suggest that miR-6794-5p-induced M2 polarization in macrophages occurs through the SOCS1/JAK1/STAT3 pathway.

**Fig. 8:**
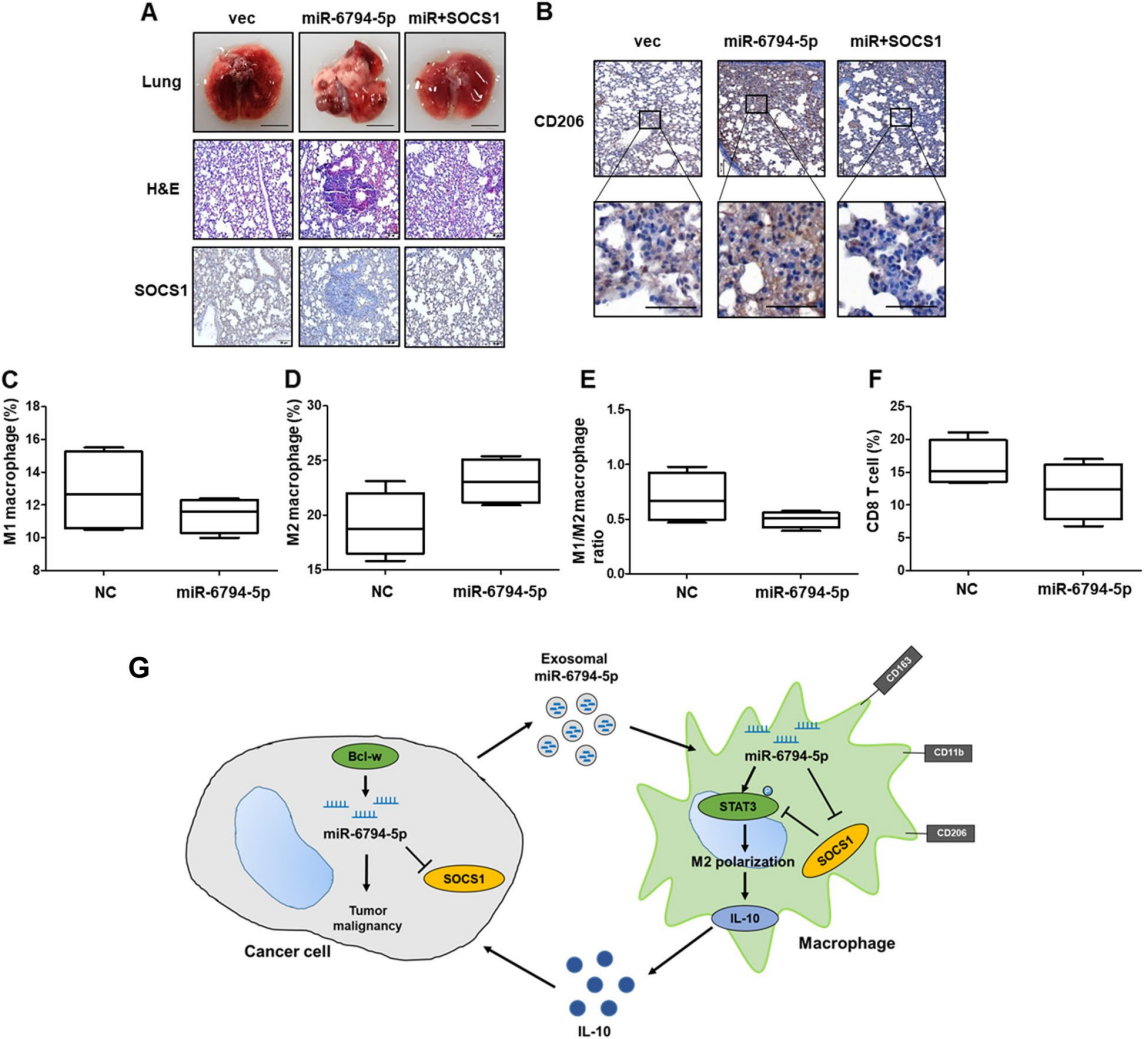
miR-6794-5p increases metastasis by inducing M2 polarization in vivo. **A**-**B** C57BL/6 mice were injected via tail vein with negative control (NC), miR-6794-5p, or miR-6794-5p + SOCS1 overexpressing LLC1 cells (*n* = 5; 5 X 10^5^ cells/mouse). Lung tissue was harvested by sacrifice at 4 weeks after cell injection. **A** Lung tissue images of each group were subjected to H&E and IHC staining with anti-SOCS1. Scale bar is 100 μm. **B** IHC staining was performed with anti-CD206 using the lung tissues of each group. Scale bars are 100 μm (top) and 50 μm (bottom). **C**-**F** Negative control (NC) and miR-6794-5p overexpressed LLC1 cells were subcutaneously injected into the right flank of C57BL/6 mice (*n* = 4; 2 X 10^5^ cells/mouse). After tumorigenesis, the expression levels of M1 macrophages, M2 macrophages and activated CD8+ T cells in the tumor tissues of the two groups were analyzed by representative flow cytometry. **C, D** Respective percentages of M1 macrophages (CD45+ F4/80+ CD11b + MHCII+ CD206- cells) and M2 macrophages (CD45+ F4/80+ CD11b + MHCII- CD206+ cells) isolated from tumor tissues developed in negative control (NC) and miR-6794-5p overexpressing mice were analyzed by flow cytometry. **E** The M1/M2 ratio was calculated based on the percentages of M1 and M2 macrophages analyzed by flow cytometry. **F** The proportion of activated CD8+ T cells (CD45+ CD8+ CD3+ CD25+ cells) isolated from tumor tissue developed in negative control (NC) and miR-6794-5p overexpressing mice was analyzed by flow cytometry. **G** Schematic diagram illustrating the mechanism of exosomal miR-6794-5p secreted from tumor cells on surrounding macrophages

An animal model was used to confirm the tumorigenicity and metastatic ability of miR-6794-5p at the cellular level. LLC1 cells overexpressing miR-6794-5p were injected into the tail vein of C57BL/6 mice; mice were harvested 3 weeks later to obtain lung tissues. The pulmonary nodules, which were increased by miR-6794-5p, were reduced by SOCS1 overexpression (Fig. 8A, top). Hematoxylin and eosin (H&E) staining confirmed that miR-6794-5p overexpression increased tumor size, which was reduced by SOCS1 overexpression (Fig. 8A, middle); SOCS1 expression was confirmed in each group (Fig. 8A, bottom). These results verified the negative relationship between miR-6794-5p and its target SOCS1. Immunohistochemistry (IHC) staining confirmed that CD206 expression, which was increased by miR-6794-5p overexpression, was decreased by SOCS1 overexpression in the lung tissues of each group (Fig. 8B). The harvested tumor tissue was analyzed by flow cytometry to confirm the subtype of macrophages around the tumor (Fig. 8C-F). The proportion of M1 macrophages, M1/M2 ratio, and CD8+ T cells decreased when miR-6794-5p was overexpressed (Fig. 8C, E, F), whereas the distribution of M2 macrophages increased (Fig. 8D). These results suggested that miR-6794-5p inhibits CD8+ T cell activity and induces M2 polarization in vivo. Taken together, these results indicate that miR-6794-5p is involved in inducing the M2 polarization of macrophages in the tumor microenvironment, leading to tumorigenicity and metastasis (Fig. 8G).

## Discussion

This study suggests the possibility of exosomes, key factors in cell-to-cell communication, as a major factor in Bcl-w-induced cancer malignancy, as identified in previous studies [9]. Cell-to-cell communication occurs through complex systems involving secreted factors or direct cell-to-cell contacts [49]. In particular, it has been reported that the oncogenic signals of exosomal miRNAs affect cancer growth, angiogenesis, metastasis, immune response, chemoresistance, and remodeling of the tumor microenvironment through interactions with local and distant cells [20, 32, 50]. Additionally, during tumor progression, exosomal miRNAs secreted from primary tumors can be transferred to non-malignant cells in the tumor microenvironment, thereby promoting heterogeneity [35, 51]. In this study, we confirmed that miR-6794-5p is highly expressed in Bcl-w-overexpressing cancer cell-derived exosomes. Using in vitro and in vivo models, we demonstrated that miR-6794-5p increased tumor progression by promoting cell migration, invasiveness, and maintenance of stemness (Fig. 2). We identified a gene, SOCS1, that is directly down-regulated by miR-6794-5p. SOCS1 is a tumor suppressor in cancer cells that downregulates cytokines by inhibiting the JAK/STAT pathway [52]. Additionally, SOCS1 forms a complex with the DNA damage-regulatory kinases ATM/ATR to increase the transcriptional activity of p53, which is involved in DNA repair, senescence, and apoptosis [52, 53]. Our study revealed that miR-6794-5p inhibits SOCS1 expression at the cellular level by directly binding to it and confirmed that SOCS1 was expressed at a lower level in plasma samples from patients with lung cancer than in normal groups (Fig. 3). As a result of KM plot analysis, low expression of SOCS1 was positively correlated with poor survival rate, supporting our results.

Furthermore, as exosomes are a major factor in cell-to-cell communication, research on exosomes as a means of communication between cancer cells and surrounding cells has been conducted. Macrophages and fibroblasts, which are important components of the tumor microenvironment, promote cancer cell proliferation, angiogenesis, and recruit immune cells that make structural and functional contributions at all stages of cancer progression by secreting growth factors, cytokines, and chemokines. Macrophages and fibroblasts have been reported as key factors in cancer malignancy [54]. Treatment of cancer cells with conditioned media from macrophages and fibroblasts overexpressing miR-6794-5p confirmed that EMT, cell mobility, invasiveness, and stem cell ability increased in the presence of conditioned media from macrophages rather than that from fibroblasts (Fig. S8). These results suggest that cancer cell-derived miR-6794-5p enhances the ability of cancer cells to interact with macrophages.

IL-10 expression was increased in macrophages affected by tumor-derived miR-6794-5p, and overexpression of IL-10 by miR-6794-5p increased cancer cell motility, invasiveness, and stemness (Figs. 5 and 6). IL-10, an anti-inflammatory cytokine, creates an immunosuppressive environment in non-small cell lung cancer (NSCLC), inducing resistance to apoptosis, angiogenesis, tumor growth and metastasis [55–57]. In addition, IL-10 expression was increased in plasma samples or TCGA datasets from patients with GBM and lung cancer (Fig. 5G, H). These results are consistent with previous reports that high serum IL-10 expression in NSCLC is associated with poor survival [58]. When U251 and A549 cells were treated with conditioned media from macrophages overexpressing miR-6794-5p, cell mobility and invasion ability increased and were restored when IL-10 was knocked down (Fig. 6). These results showed that IL-10 induced by miR-6794-5p overexpression in macrophages induced the malignant transformation of cancer cells.

Macrophage polarization refers to the process of generating a characteristic phenotype in response to stimuli. Macrophages are divided into classically activated macrophages (M1), which secrete inflammatory cytokines and chemokines, and alternatively activated macrophages (M2), which provide an immunosuppressive microenvironment for tumor development [59, 60]. M1 macrophages promote an inflammatory response against invading pathogens and tumor cells, whereas M2 macrophages promote tissue repair and tumor progression via an immunosuppressive response [61]. Our results showed that U251- and A549-derived miR-6794-5p increased the expression of the M2 macrophage markers CD163, CD206, and CD11b, and the product of M2 macrophage IL-10 in THP-1-derived macrophages by activating the JAK1/STAT3 pathway (Fig. 7). This result is supported by a report that the activation of the JAK/STAT3 pathway is involved in the M2 polarization of macrophages [62]. In animal models, when compared with the mice in the negative control, M2 macrophages increased and the proportion of activated CD8+ T cells decreased in mice overexpressing miR-6794-5p (Fig. 8D, F). These results confirmed that miR-6794-5p increased the M2 polarization of macrophages, inhibited CD8+ T cell function, and ultimately promoted tumorigenicity.

Our study analyzed the cause of tumor malignancy induced by intratumoral Bcl-w overexpression from the perspective of the communication between macrophages and cancer cells in the tumor microenvironment. These results revealed that exosomal miR-6794-5p induces tumorigenicity by suppressing the expression of SOCS1, a target factor. In addition, tumor-derived exosomal miR-6794-5p promoted M2 polarization and the secretion of IL-10 by macrophages by activating the JAK1/STAT3 pathway, ultimately promoting tumor malignancy. Meanwhile, because exosomes are secreted into body fluids from various cells, including cancer cells, exosomal miRNA analysis can be used as a useful biomarker for cancer diagnosis and treatment using liquid biopsy in the future, therefore many studies are being conducted in this regard [63]. Although miRNAs are limited by their low stability, exosomal miRNAs are protected from RNase degradation owing to lipid bilayer protection. As this may increase their stability [32], they have high potential as biomarkers for monitoring cancer progression and therapeutic efficacy. This study reveals the mechanism of action of miR-6794-5p as an onco-miRNA in cancer cells and suggests its potential as a biomarker for diagnosis and treatment.

## Conclusion

In summary, our study demonstrated how tumor-derived miR-6794-5p is involved in tumor malignancy by remodeling the tumor microenvironment. MiR-6794-5p, which was upregulated by Bcl-w which is highly expressed in tumor cells, increased tumorigenicity by suppressing SOCS1 expression. Additionally, tumor-derived exosomal-miR-6794-5p translocates to macrophages in the surrounding tumor microenvironment and activates the JAK1/STAT3 pathway by inhibiting SOCS1 expression, resulting in M2 polarization of macrophages. M2 polarization of macrophages promotes the secretion of IL-10, which ultimately leads to tumor malignancy and metastasis. As such, tumor-derived miR-6794-5p promotes tumor malignancy by inducing an immunosuppressive environment through macrophage M2 polarization, suggesting its potential as a biomarker for diagnostic and therapeutic development.

### Supplementary Information

**Supplementary Material 1.**

## Data Availability

The TCGA data used in the analysis was provided by UCSC Xena [https://xenabrowser.net/datapages/]. The Km plot used in the analysis was provided by Kaplan-Meier plotter [https://www.kmplot.com/]. The raw data of the miRNA microarray was uploaded to Gene Expression Omnibus (GEO) within NCBI, a public repository (GSE260909).

## Abbreviations

miRNA: MicroRNA
IL-10: Interleukin 10
GBM: Glioblastoma multiforme
DMA: Dimethyl amiloride
EMT: Epithelial–mesenchymal transition
TEM: Transmission electron microscope
exo: Exosome
LLC1: Lewis lung cancer cell line
KM plot: Kaplan-meier plot
TCGA: The cancer genome atlas program
MΦ: Macrophage
VEGF: Vascular endothelial growth factor
EGF: Epidermal growth factor
H&E: Hematoxylin and eosin
IHC: Immunohistochemistry
NSCLC: Non-small cell lung cancer
cDNA: Complementary DNA
