# Supplementary material for: Tumor-derived miR-6794-5p enhances cancer growth by promoting M2 macrophage polarization
Source: Cell Commun Signal. 2024 Mar 23;22:190. doi: 10.1186/s12964-024-01570-5 (PMC10960442; doi:10.1186/s12964-024-01570-5)
Supplement: Supplementary file 1 — Supplementary Material 1. [file 12964_2024_1570_MOESM1_ESM.pdf]

Fig. S1

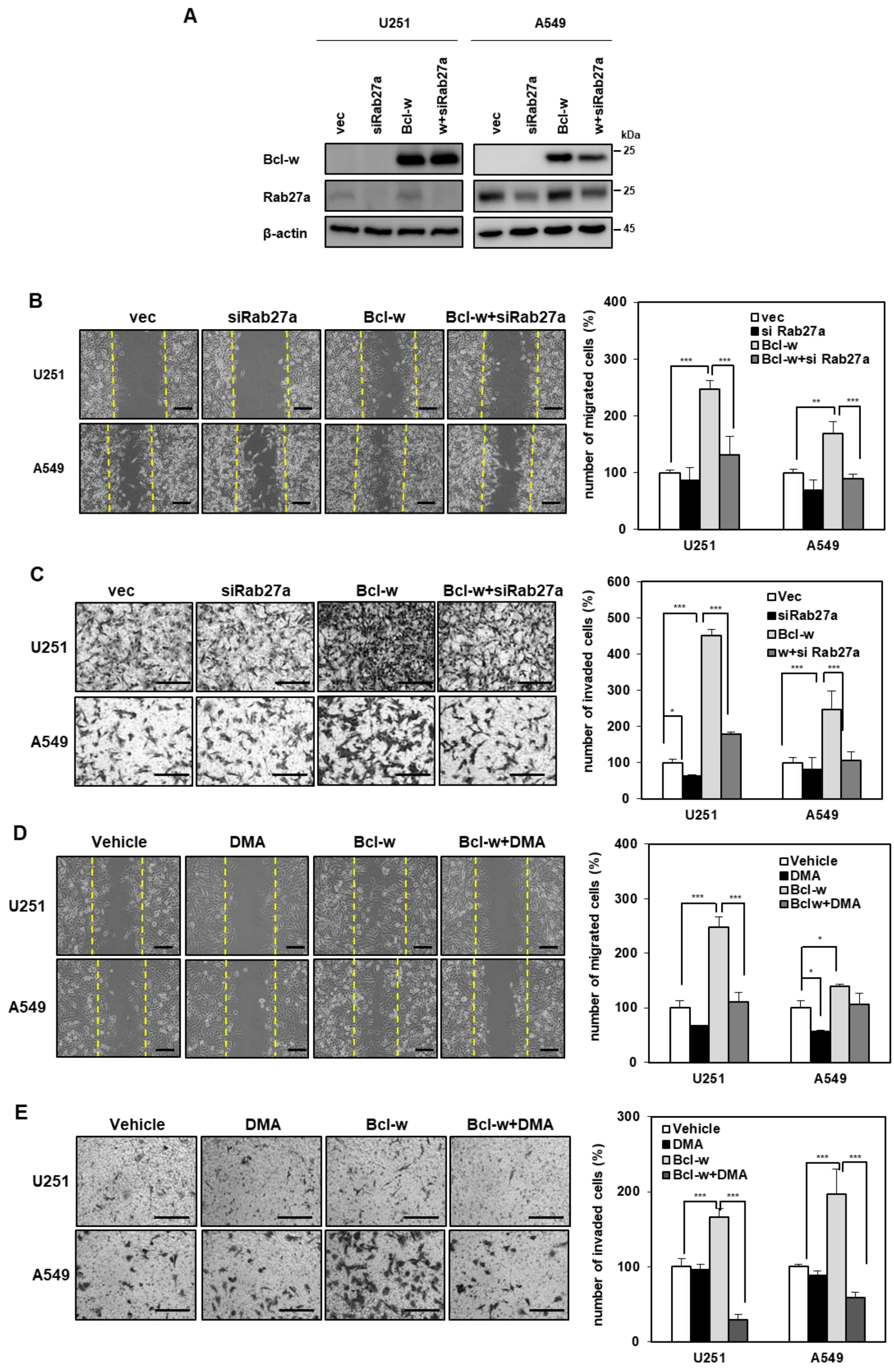

Fig. S1 Tumor-derived exosomes upregulate Bcl-w-induced tumorigenicity. **A-E** To investigate the relationship between the oncogenic gene Bcl-w and exosome secretion in cancer cells, U251 and A549 cells were transfected with an empty vector or a Bcl-w overexpression vector. **A** Indicated cells were transfected with Bcl-w or Rab27a siRNA, and the expression of Bcl-w or Rab27a in lysates of the indicated cells was confirmed by western blot analysis. β-actin was used for normalization in this assay. Wound healing (**B**) and matrigel coated invasion (**C**) assays were subjected on the indicated cells. **D,E** After treating empty vector or Bcl-w overexpressing cells with or without DMA (dimethyl amiloride, an exosome release inhibitor) (25 ug/ml) for 4 h, wound healing (**D**) and matrigel invasion (**E**) assays were subjected on the indicated cells. Scale bar is 200 μm. The data are presented as the mean ± S.D. after triplicate. $^*P < 0.05$; $^{**}P < 0.01$; $^{***}P < 0.001$. Student`s t-test.

Fig. S2

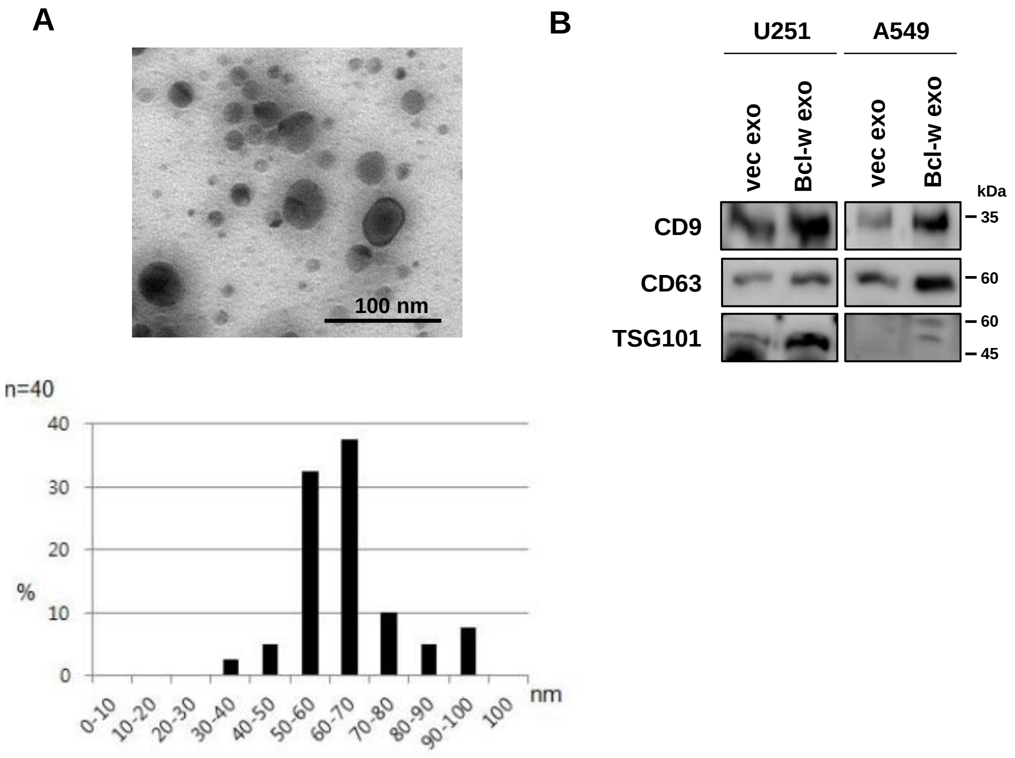

Fig. S2 Identification of exosomes isolated from conditioned media of Bcl-w overexpressed cancer cells. (A) Representative Transmission Electron Microscope (TEM) images were shown (top) and distribution of exosome size was quantified (bottom). Scale bar is 100 nm. (B) The expression of exosome markers CD9, CD63, and TSG101 in the isolated exosomes was confirmed by western blot analysis.

Fig. S3

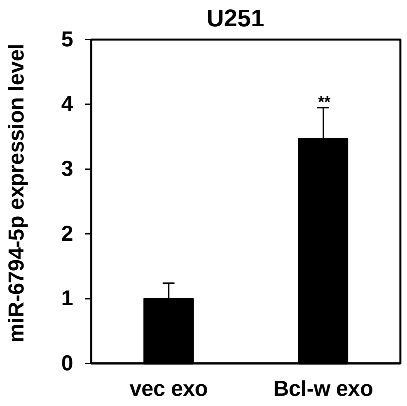

Fig. S3 Expression of miR-6794-5p is increased in exosomes secreted from overexpressing Bcl-w U251 cells**.** The level of miR-6794-5p was examined by isolating exosomes from the conditioned media of Bcl-w overexpressing U251 cells by qRT-PCR. The values were normalized to U6. The data are presented as the mean ± S.D. after triplicate. **P < 0.01. Student`s t-test.

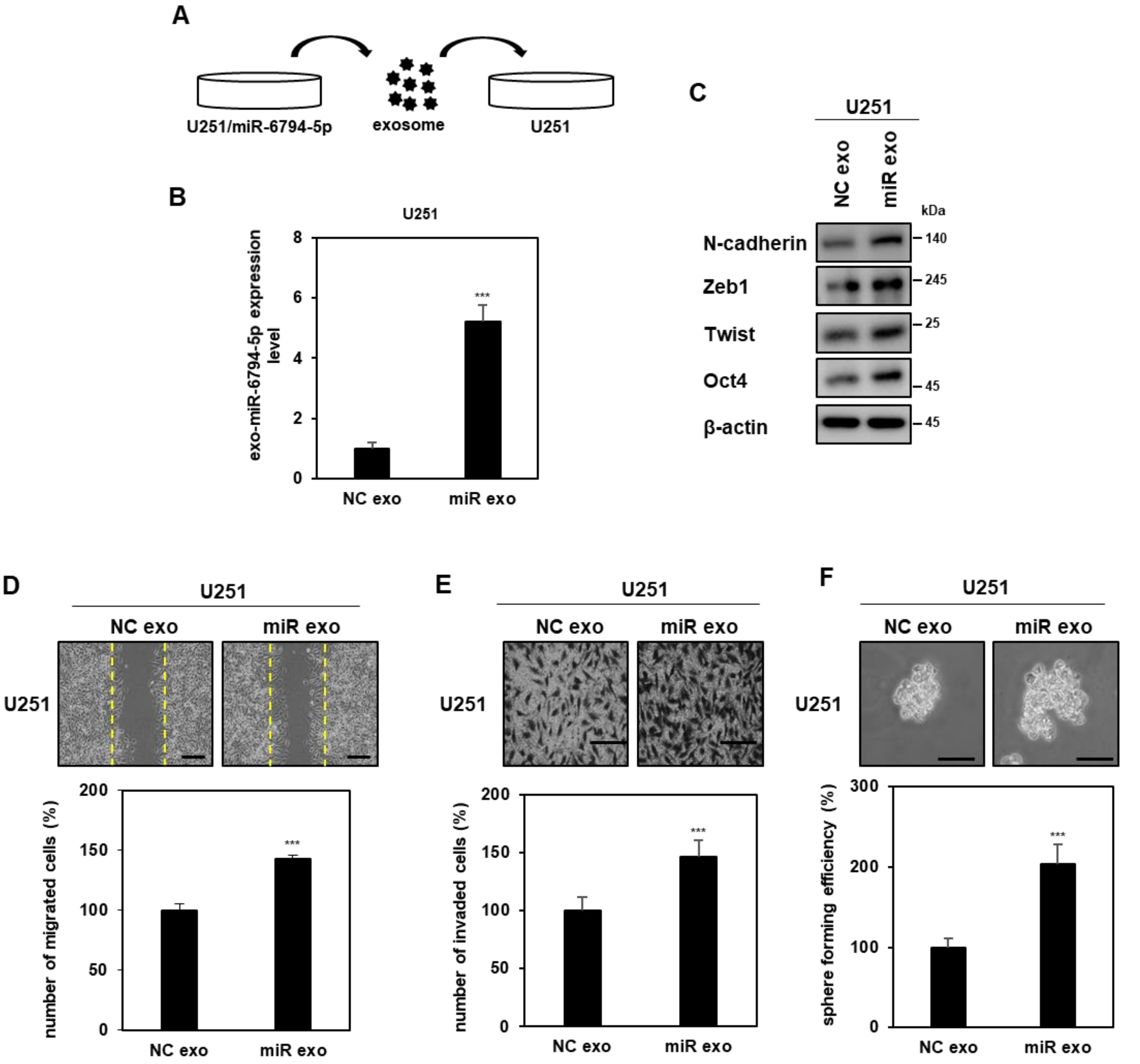

Fig. S4 Tumor-derived exosomal miR-6794-5p induces aggressiveness of surrounding cancer cells. **A** Scheme to investigate the effect of exosomes isolated from conditioned media of overexpressing miR-6794-5p U251 cells on the malignancy of surrounding cancer cells. **B** The expression of miR-6794-5p in isolated exosomes was confirmed by qRT-PCR. The values were normalized to U6. **C-F** After treating U251 cells with isolated exosomes, expression of mesenchymal and stem cell marker (**C**), wound healing ability (**D**), matrigel coating invasiveness (**E**), and sphere formation ability **(F**) were measured by western blot, wound healing, matrigel coated invasion, and sphere formation assays, respectively. β-actin was used for normalization of western blot analysis. Scale bar is 200 μm. The data are presented as the mean ± S.D. after triplicate. ***P < 0.001. Student`s t-test.

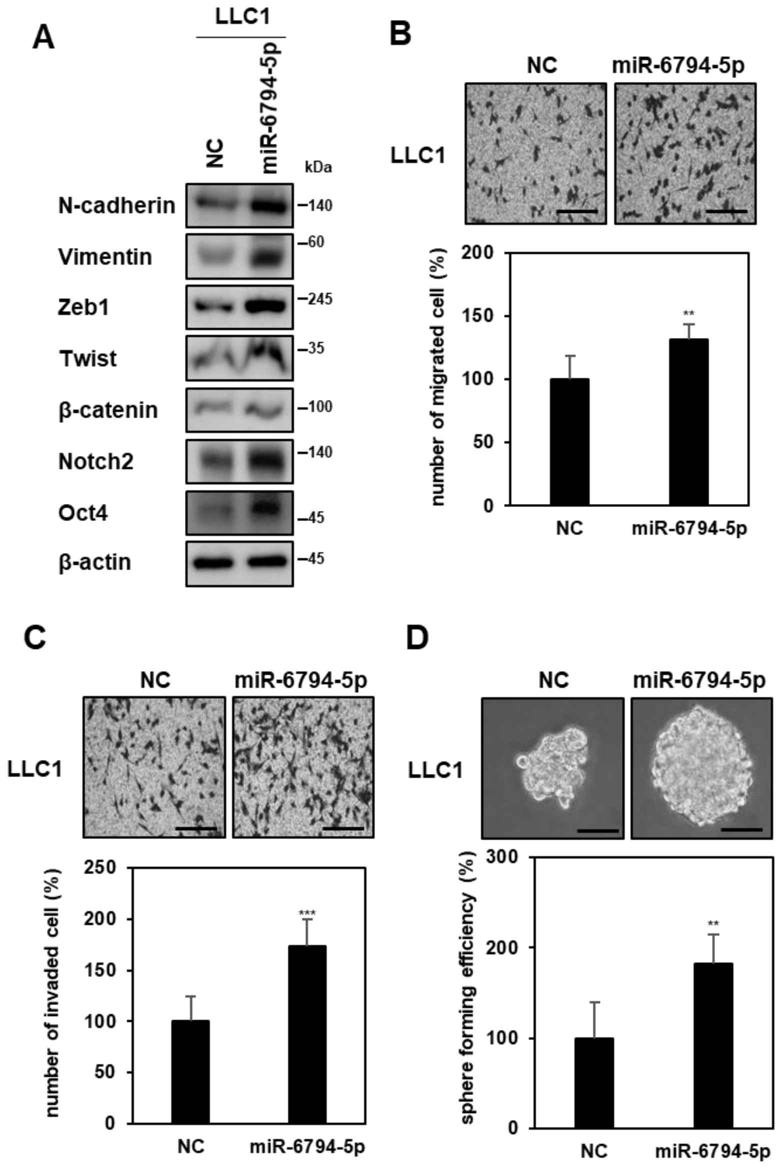

Fig. S5 miR-6794-5p also promotes EMT, migratory ability, invasiveness, and stemness maintenance in LLC1 cells. **A-D** After LLC1 cells were transfected with either negative control (NC) or miR-6794-5p mimic, expression of mesenchymal and cancer stem-like cell markers (**A**), migratory ability (**B**), invasiveness (**C**), and sphere formation ability (**D**) were measured by western blot, transwell migration, matrigel coated invasion, and sphere formation assays in the indicated cells, respectively. β-actin was used for normalization in western blot analysis. Scale bar is 200 μm. The data are presented as the mean ± S.D. after triplicate. **P < 0.01; ***P < 0.001. Student`s t-test.

Fig. S6

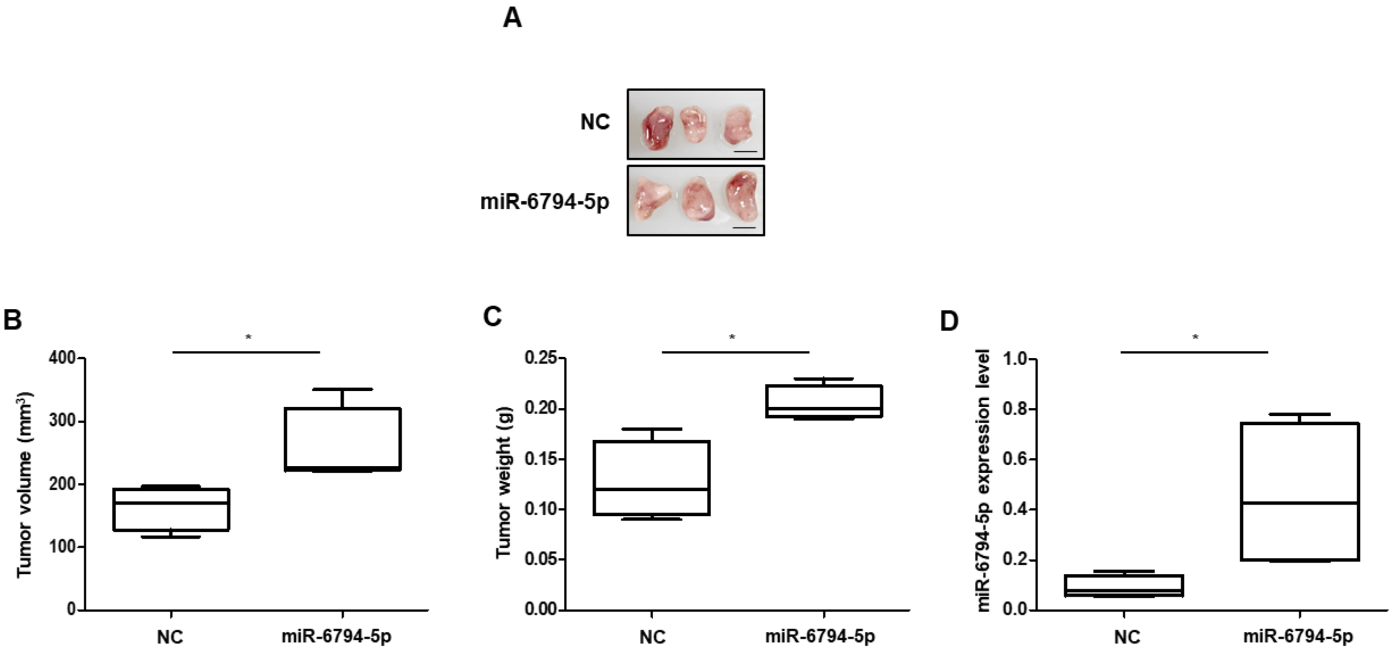

Fig. S6 miR-6794-5p induces tumorigenicity in vivo. **A-D** Negative control (NC) or miR-6794-5p overexpressing LLC1 cells were injected subcutaneously into the right flank of C57BL/6 mice (n = 4; 2 × 10^5 cells/mouse), respectively. After harvesting mice on 14days, whole tumor images (**A**), tumor volume (**B**), and tumor weights (**C**) of the negative control (NC) and miR-6794-5p groups were obtained. **D** The expression of miR-6794-5p in the plasma of mice was analyzed by qRT-PCR. *P < 0.05. Student`s t-test.

Fig. S7

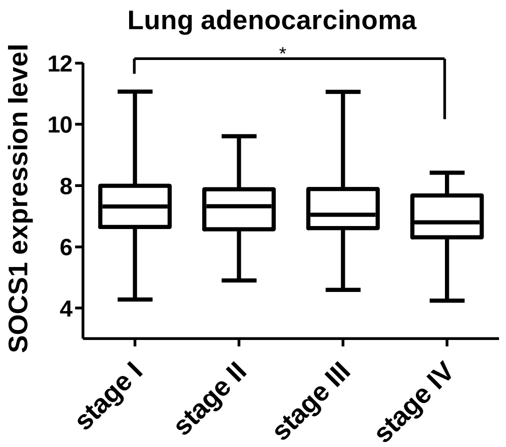

Fig. S7 SOCS1 expression is downregulated in high stages of lung adenocarcinoma. Using the TCGA dataset, the expression pattern of SOCS1 was analyzed in stage I to IV patients with lung adenocarcinoma. *P < 0.05. Student`s t-test.

Fig. S8

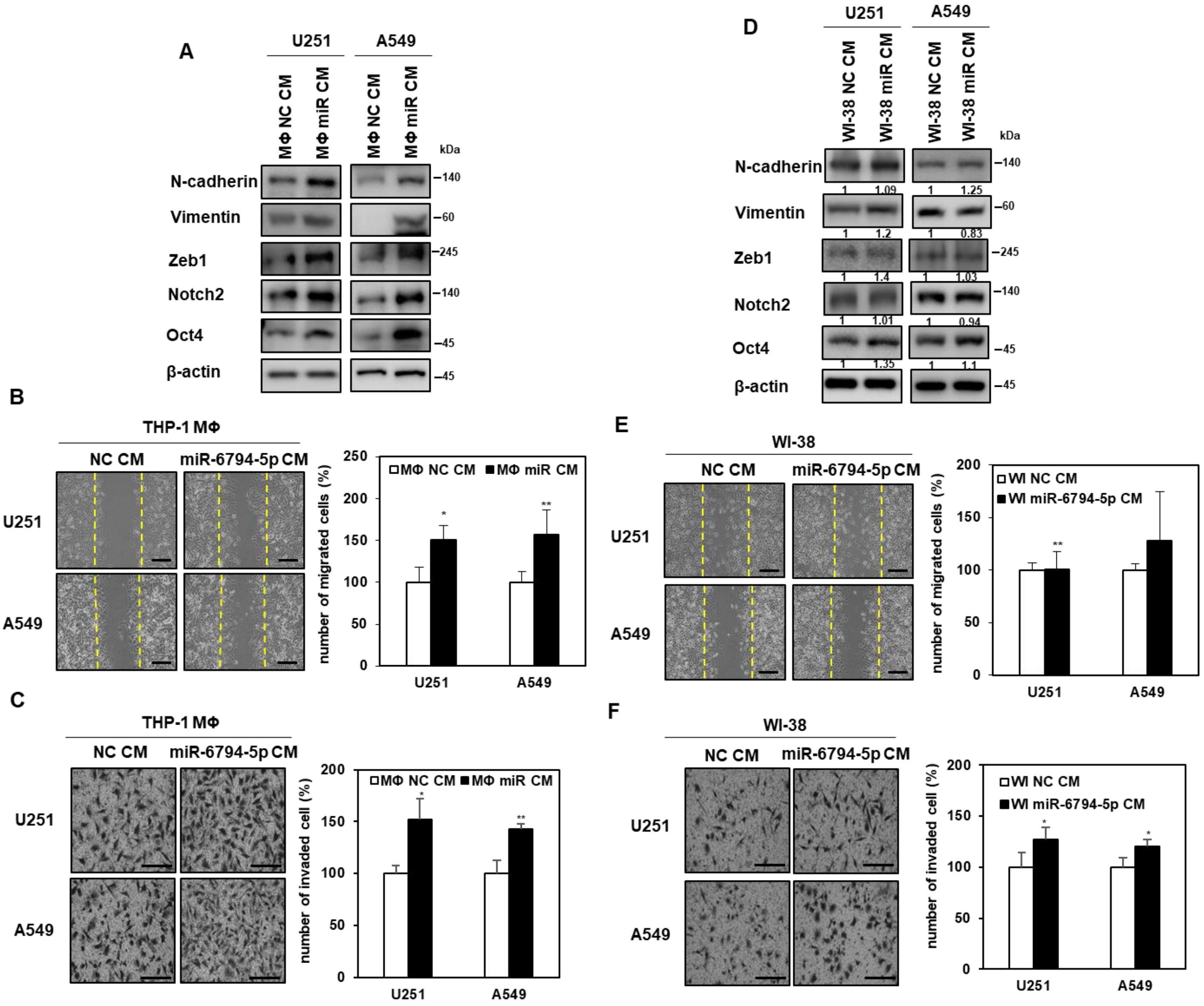

Fig. S8 Tumor-derived miR-6794-5p increases tumor malignancy through interaction with macrophages. **A-C** THP-1 was treated with 100nM Phorbol 12-myristate 13-acetate (PMA) for 24 hours to differentiate into macrophages. After transfecting THP-1 derived macrophages (**A-C**) and human fibroblasts WI-38 (**D-F**) with NC or miR-6794-5p mimic, the conditioned media were collected 48 hours later. After treating U251 and A549 cells with the conditioned media obtained from the THP-1 derived macrophages and WI-38 cells, respectively, western blot (**A,D**), wound healing (**B,E**), and matrigel coating invasion (**C,F**) assays were performed. β-actin was used for normalization in western blot analysis. Scale bar is 200 μm. The data are presented as the mean ± S.D. after triplicate. *P < 0.05; **P < 0.01; ***P < 0.001. Student`s t-test.

Fig. S9

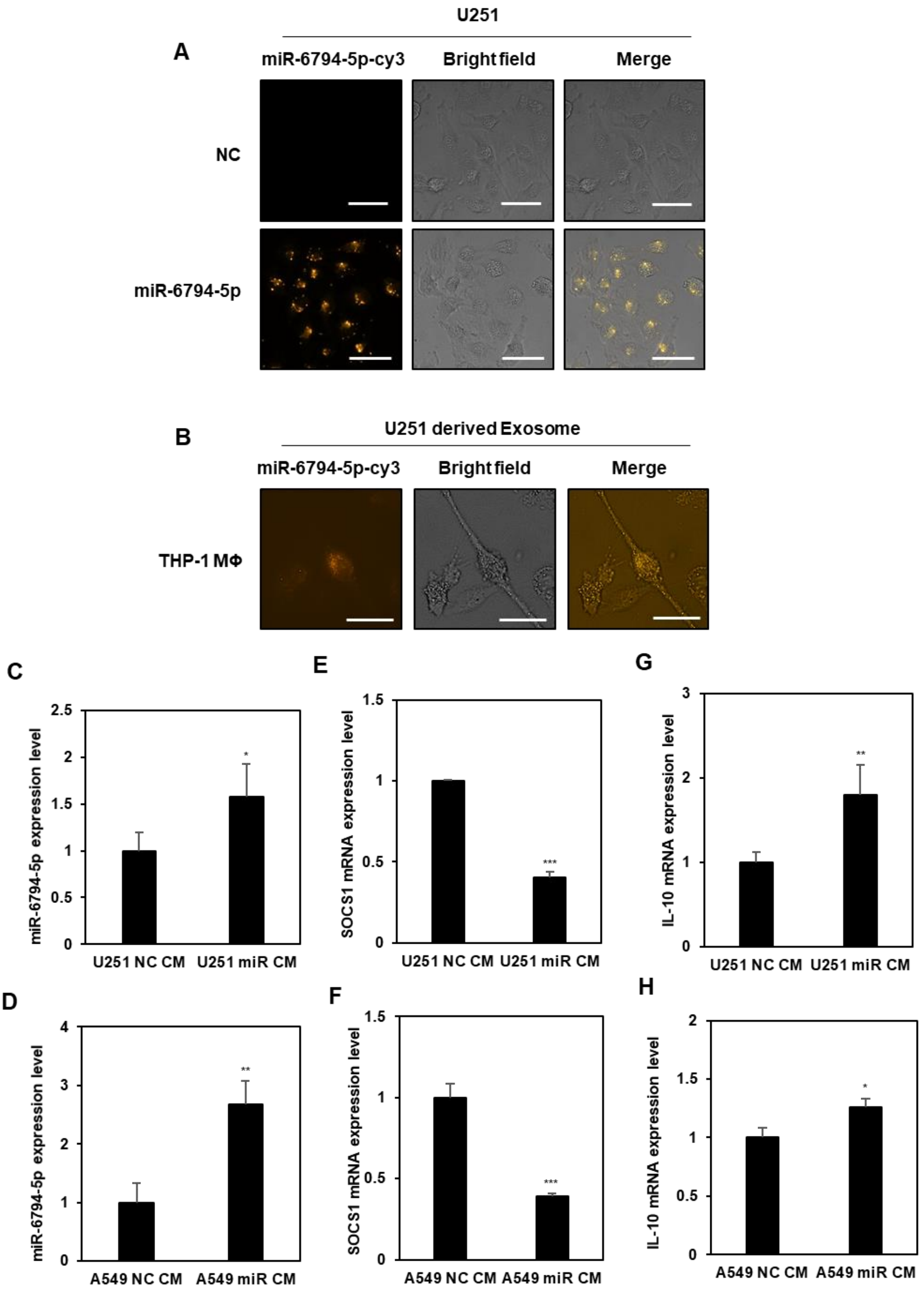

Fig. S9 Tumor-derived miR-6794-5p is delivered to THP-1-derived macrophages to increase IL-10 expression. **A** Fluorescence of U251 cells transfected with cy3-tagged miR-6794-5p was observed under INCELL2000 analyzer. Scale bar is 60 µm. **B** Exosomes were isolated from conditioned media of U251 overexpressing cy3-tagged miR-6794-5p. The isolated exosomes were treated with THP-1-derived macrophages and observed under a INCELL2000 analyzer. Scale bar is 30 µm. **C-H** After treating THP-1-derived macrophages with the conditioned media of U251 or A549 in which miR-6794-5p was overexpressed, the expression level of miR-6794-5p (**C,D**), SOCS1 (**E,F**), and IL-10 (**G,H**) were measured by qRT-PCR analysis. The values were normalized to U6. The data are presented as the mean ± S.D. after triplicate. *P < 0.05; **P < 0.01; ***P < 0.001. Student`s t-test.

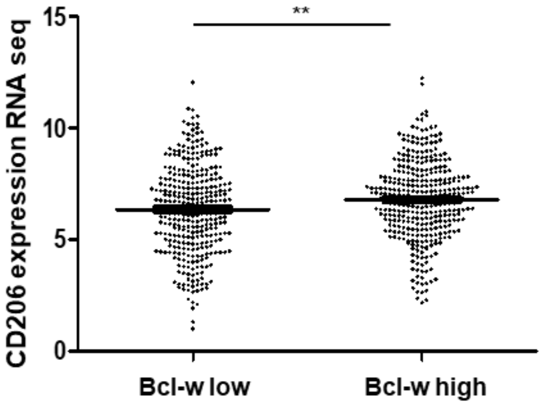

Fig. S10 CD206 expression is slightly elevated in patients with high Bcl-w expression. Using the TCGA database, the results of comparing the expression of CD206 in GBM patients with low (n = 327) or high (n = 352) expressed Bcl-w were displayed as a scatter plot. **P < 0.01. Student`s t-test.
